# Global, regional, and national burden of motor neuron disease in adults aged 65 years and older from 1990 to 2021 and forecast to 2040

**DOI:** 10.3389/fneur.2025.1641887

**Published:** 2025-07-24

**Authors:** Quanfeng Zhao, Bin Zhang, Xiulan Chen, Peishu Fu, Yang Yang, Qian Wang

**Affiliations:** ^1^Department of Pharmacy, Southwest Hospital, Third Military Medical University (Army Medical University), Chongqing, China; ^2^Department of Neurology, Guangdong Neuroscience Institute, Guangdong Provincial People's Hospital (Guangdong Academy of Medical Sciences), Southern Medical University, Guangzhou, China; ^3^Guangdong Provincial People’s Hospital, Guangdong Academy of Medical Sciences, Southern Medical University, Guangzhou, Guangdong, China; ^4^Department of Pharmacology, Chongqing Health Center for Women and Children, Chongqing, China; ^5^Department of Pharmacology, Women and Children’s Hospital of Chongqing Medical University, Chongqing, China; ^6^NHC Key Laboratory of Birth Defects and Reproductive Health, Chongqing, China

**Keywords:** motor neuron disease, amyotrophic lateral sclerosis, disease burden, elderly, socio-demographic index

## Abstract

**Aim:**

Given the significant social burden of motor neuron disease (MND) among elderly patients (aged ≥ 65 years) and the lack of detailed research on its epidemiological characteristics, this study aims to elucidate the temporal trends and distributional characteristics of MND in the elderly from 1990 to 2021, as well as to forecast its future burden.

**Methods:**

The age-standardized rates (ASR) and absolute numbers of MND-related incident, prevalent, death, and disability-adjusted life years (DALYs) among older patients (aged ≥ 65 years) globally were derived from the Global Burden of Disease (GBD) study from 1990 to 2021. The data were derived by gender, age group and geographic region. An estimated annual percentage change (EAPC) was estimated to represent temporal trends, and a Bayesian Age-Period-Cohort model was used to forecast the future burden of elderly MND.

**Results:**

In 2021, the global ASRs of incidence, prevalence, mortality, and DALYs for elderly MND were 3.63 (95% uncertainty intervals [UI], 2.95–4.36), 11.45 (95% UI, 8.69–14.88), 3.28(95% UI, 2.90–3.61), and 59.92 (95% UI, 53.94–65.53), respectively. Elderly patients those were from high socio-demographic index (SDI) region, as well as males, exhibited the highest burden. From 1990 to 2021, the global ASRs of elderly MND increased, with EAPCs of 0.43 (95% confidence interval [CI], 0.38–0.49), 0.58 (95% CI, 0.48–0.68), 0.90 (95%CI, 0.75–1.06), and 0.84 (95% CI, 0.71–0.96), respectively. Positive correlations were found between sociodemographic index and the burden of elderly MND. Health inequalities were evident across 204 countries and regions, with the inequality slope index raised from 23.46 (95% CI: 18.52–28.40) in 1990 to 80.70 (95% CI: 65.07–96.32) in 2021. Compared to the figures observed in 2021, our forecasts indicate a continued rise in the burden of elderly MND up to 2040, with the projected ASIR expected to reach 3.15 (95% UI, 2.28–4.01) and the ASMR anticipated to be 3.32 (95% UI, 2.11–4.55).

**Conclusion:**

The burden of MND among elderly patients is substantial, particularly in high SDI region and among males. From 1990 to 2021, the global burden of elderly MND has exhibited an increasing trend. The burden of elderly MND varies significantly across the world, necessitating more targeted screening strategies and preventive measures to address the issue of elderly MND.

## 1 Introduction

Motor neuron disease (MND), also known as amyotrophic lateral sclerosis (ALS) in the United Kingdom (1) and often referred to interchangeably with ALS in the United States, is a progressive neurodegenerative disease characterized by the selective death of motor neurons in the motor cortex and spinal cord. This degeneration can lead to limb paralysis, dysarthria, dysphagia, and even respiratory failure (2). Unfortunately, effective treatments for MND remain elusive, and the prognosis is generally poor, with a median survival rate of only 3–5 years (3, 4). MND not only shortens patients’ life expectancy but also diminishes their quality of life and imposes significant healthcare costs (5). A review revealed that the standardized total annual cost per MND patient in the United State was approximately $70,000 (6). Although MND is relatively rare in the natural populations, its high lethality and serious physical injuries have heightened global awareness of the disease, particularly following the widespread attention generated by the Ice Bucket Challenge (7).

People of any age can develop MND, but MND usually affects people over the age of 50, especially elder adults more than 65 years are particularly susceptible to MND (8–10). Managing elderly adults (aged ≥ 65 years) with MND is more complex, given multiple comorbidities and diminished physical function. Moreover, the number of prevalent cases is soaring, primarily driven by population aging. In recent years, the epidemiology of MND has been reported in many parts of the world (10). However, a comprehensive picture of the MND burden among patients aged 65 years and older remains unknown at the global, regional, and national levels. Epidemiological data are essential for implementing effective disease prevention measures and allocating health resources to address this significant but under-reviewed health challenge.

The Global Burden of Disease (GBD) database compiles data from a wide array of sources, including national and international health surveys, hospital records, clinical studies, death records and registries. The 2021 GBD database encompasses the disease burden of 371 diseases and injuries across 204 countries and regions, as well as 811 sub-national regions for the period 1990 to 2021 (11). This provides a unique platform for comparing the magnitude of disease across different age groups, genders, regions, and time frames.

In this study, we aimed to provide a comprehensive assessment of the global incidence, prevalence, mortality, and disability-adjusted life years (DALYs) of MND among elderly patients (aged ≥ 65 years) from 1990 to 2021, and to forecast trends up to 2040. We hope that these findings will contribute to an in-depth understanding of the global distribution and extent of the MND burden among elderly patients, and help to establish disease control and prevention strategies in various countries and regions.

## 2 Methods

### 2.1 Study data

Data in this study were obtained from the GBD 2021 results (available from https://vizhub.healthdata.org/gbd-results/). The definition of MND was based on the international classification of diseases, 10th version (ICD-10), with the code of G12 (11). This study collected data on the number of cases and age-standardized rates (ASRs) of MND patients aged ≥ 65 years by sex, region, and country from 1990 to 2021. The ASR metrics encompassed the age-standardized incidence rate (ASIR), age-standardized prevalence rate (ASPR), age-standardized mortality rate (ASMR), and age-standardized DALYs rate (ASDR), and all rates were calculated per 100,000 population, represented as estimated values with 95% uncertainty intervals (UIs). The methodology of ASR is mainly based on previous studies (12, 13). The age groups were stratified into seven categories: 65–69 years, 70–74 years, 75–79 years, 80–84 years, 85–89 years, 90–94 years, and those aged 95 years and above. The socio-demographic development of a country or region is quantified by the socio-demographic index (SDI), a comprehensive indicator that reflects the social and economic conditions influencing health. The SDI were defined as the total fertility rate of the population under 25 years old, the average education level of the population aged 15 and above, and the distribution of per capita income. Then the 204 countries and regions were categorized into five regions according to their SDI, namely low, low-middle, middle, high-middle and high region. Additionally, the estimated annual percentage change (EAPC) was employed to evaluate trends in changes. The definitions of the measurements and metrics used were detailed in Supplementary Appendix 1.

### 2.2 Age-period-cohort model

The Age-period-cohort (APC) model is a reliable statistical approach for elucidating the effects of biological aging (age effect), social change (period effect), and generational exposure (cohort effect) on disease’s burden. It has emerged as a powerful tool for understanding how these factors contribute to changes in epidemiological measures over time. In this model, the age effect incorporates changes in disease risk associated with the natural aging process. The period effect evaluates how risks for all age groups changes over a specific period of time, which is usually due to factors such as economic conditions or public health measures. Additionally, the cohort effect examines the distinct experiences of individuals born in the same time period, which may be shaped by the social, economic, and health-related events of their era. These factors collectively contribute to explaining the disparities in disease risk observed across different age groups. For a comprehensive understanding of the related methodological details, readers are referred to prior publications (14, 15). The strengths of this methodology lie in its ability to enhance the accuracy of trend forecasting and policy assessment by pinpointing the drivers of health disparities.

### 2.3 Cross-country inequality analysis

Cross-country Inequality Analysis is a methodology employed to evaluate disparities in disease burden indicators across different countries, thereby identifying inequalities in the allocation of health resources and health outcomes. The Slope Index of Inequality (SII) and the Concentration Index, as defined by the World Health Organization, are commonly utilized metrics. The SII is calculated by a robust weighted regression method based on the health indicators by regressing them on country/region SDI rankings (16). The SII is the difference in predicted health indicator values between the highest and lowest SDI rankings. It represents the difference in predicted health indicator values between the highest and lowest SDI rankings, reflecting absolute inequality across socioeconomic strata. In contrast, the Concentration Index is derived from the Lorenz Concentration Curve and serves as a measure of relative inequality (16). A negative SII value suggests that the health burden is disproportionately concentrated in low SDI countries, and similarly, a negative CI value indicates a higher concentration of the health burden in low SDI countries.

### 2.4 Bayesian age-period-cohort models for projections

This study utilized the Bayesian Age-Period-Cohort (BAPC) model to predict future disease burdens (17), given its capability to manage complex, high-dimensional, and sparse data often encountered in large-scale epidemiological studies like the GBD 2021. A notable advantage of the BAPC model is its use of the Integrated Nested Laplace Approximation method to approximate marginal posterior distributions. The model’s flexibility and robustness in processing time-series data render it particularly suitable for long-term disease burden projections. It has been widely validated and applied in epidemiological studies, especially in studies involving age-structured population data and complex cohort effects (16). The methodology of BAPC is mainly based on previous studies (18), and the specific steps are detailed in Supplementary Appendix 2.

All reported *p*-values were two-sided, and statistical significance was set at *p* < 0.05. Data analysis was conducted using R software (version 4.2.0), and results were visualized with the ‘ggplot2’ package.

## 3 Results

### 3.1 Global trends

The number of cases and ASRs of MND among the elderly population (aged more than 65 years) in 1990 and 2021 were listed in Tables 1, 2 and Supplementary Tables S1, S2.

**Table 1:** Incidence of MND in patients aged 65 years and older and related EAPCs from 1990 to 2021.

Characteristics	Cases		Age-standardized incidence rate (per 100,000)		EAPCs
	1990	2021	1990	2021	1990–2021
Global	10710.76(8415.06,13191.29)	27817.15(22596.79,33365.61)	3.28(2.57,4.05)	3.63(2.95,4.36)	0.43(0.38,0.49)
Gender					
Male	5134.30(4062.40,6272.10)	14603.38(11935.67,17397.49)	3.70(2.92,4.53)	4.25(3.47,5.07)	0.58(0.52,0.64)
Female	5576.45(4346.37,6918.44)	13213.77(10659.11,15991.63)	2.99(2.33,3.72)	3.15(2.54,3.81)	0.25(0.20,0.29)
SDI					
High SDI	7480.85(6015.96,8925.10)	18746.64(15481.91,21954.49)	7.14(5.74,8.52)	9.22(7.62,10.80)	0.93(0.87,0.98)
High-middle SDI	1727.66(1260.95,2274.60)	4748.78(3658.27,5908.55)	2.07(1.50,2.73)	2.59(2.00,3.23)	0.93(0.79,1.06)
Low SDI	180.61(105.57,278.70)	367.66(220.34,553.98)	1.15(0.67,1.79)	1.02(0.61,1.55)	−0.40(−0.50, −0.30)
Low-middle SDI	410.62(243.48,620.38)	1085.59(712.05,1541.44)	0.94(0.56,1.43)	0.95(0.62,1.36)	0.14(0.07,0.22)
Middle SDI	902.50(554.34,1335.42)	2846.88(2007.68,3858.50)	1.17(0.72,1.74)	1.24(0.87,1.68)	0.35(0.27,0.43)
Regions					
Andean Latin America	19.28(12.15,28.25)	88.84(66.05,114.57)	1.20(0.75,1.76)	1.77(1.31,2.28)	1.61(1.50,1.72)
Australasia	266.40(221.20,311.31)	842.56(698.19,989.60)	11.87(9.85,13.89)	16.04(13.30,18.84)	1.07(0.98,1.16)
Caribbean	38.37(25.38,53.84)	126.33(99.47,155.24)	1.69(1.12,2.39)	2.65(2.09,3.26)	1.65(1.54,1.77)
Central Asia	36.31(20.65,57.08)	54.41(31.54,84.07)	1.05(0.60,1.66)	0.93(0.54,1.44)	−0.42(−0.49, −0.34)
Central Europe	194.88(127.41,275.12)	504.33(381.16,638.46)	1.48(0.96,2.09)	2.25(1.70,2.86)	1.54(1.48,1.60)
Central Latin America	85.84(56.15,121.31)	465.76(359.37,582.27)	1.33(0.87,1.88)	2.18(1.68,2.73)	1.94(1.84,2.04)
Central Sub-Saharan Africa	18.96(10.73,30.18)	40.49(22.94,63.73)	1.36(0.77,2.18)	1.24(0.70,1.96)	−0.34(−0.45, −0.23)
East Asia	847.40(524.06,1246.60)	2097.82(1322.92,3047.60)	1.25(0.77,1.86)	1.04(0.65,1.51)	−0.72(−0.86, −0.57)
Eastern Europe	239.92(143.53,362.68)	565.38(411.55,742.82)	1.05(0.62,1.60)	1.67(1.21,2.20)	2.08(1.88,2.28)
Eastern Sub-Saharan Africa	74.83(41.92,118.18)	138.97(78.39,218.69)	1.47(0.83,2.34)	1.23(0.70,1.95)	−0.63(−0.76, −0.50)
High-income Asia Pacific	624.86(476.62,782.46)	2212.10(1734.85,2695.47)	3.54(2.69,4.44)	4.83(3.78,5.89)	1.19(1.11,1.27)
High-income North America	3063.09(2475.35,3636.84)	7937.54(6759.31,9084.67)	8.85(7.15,10.52)	12.41(10.57,14.20)	1.17(1.05,1.29)
North Africa and Middle East	150.74(97.03,217.10)	477.88(339.02,640.26)	1.21(0.77,1.75)	1.38(0.97,1.86)	0.52(0.47,0.57)
Oceania	2.40(1.50,3.48)	4.86(2.94,7.24)	1.20(0.75,1.76)	0.99(0.60,1.49)	−0.77(−0.89, −0.65)
South Asia	304.28(178.17,462.06)	913.04(570.69,1337.51)	0.77(0.45,1.17)	0.76(0.47,1.12)	0.07(−0.01,0.14)
Southeast Asia	182.63(105.11,284.28)	422.34(252.27,636.39)	1.00(0.58,1.56)	0.83(0.50,1.26)	−0.61(−0.73, −0.50)
Southern Latin America	106.46(74.43,142.90)	346.10(270.93,423.30)	2.57(1.79,3.46)	4.27(3.35,5.23)	1.57(1.40,1.73)
Southern Sub-Saharan Africa	23.79(13.66,37.35)	43.08(24.90,67.32)	1.17(0.67,1.85)	1.02(0.58,1.60)	−0.49(−0.63, −0.35)
Tropical Latin America	137.06(97.80,181.42)	785.62(639.61,934.04)	1.90(1.35,2.53)	3.52(2.87,4.19)	2.36(2.19,2.52)
Western Europe	4223.05(3374.74,5065.72)	9618.31(7663.25,11517.63)	7.55(6.04,9.06)	10.84(8.65,12.97)	1.29(1.23,1.34)
Western Sub-Saharan Africa	70.21(42.49,106.49)	131.40(81.24,196.76)	1.08(0.65,1.66)	0.99(0.60,1.50)	−0.29(−0.39, −0.19)

MND, motor neuron disease; SDI, socio-demographic index; EAPCs, estimated annual percentage changes.

**Table 2:** Mortality of MND in patients aged 65 years and older and related EAPCs from 1990 to 2021.

Characteristics	Cases		Age-standardized mortality rate (per 100,000)		EAPCs
	1990	2021	1990		1990
Global	8349.77(7811.48,8729.57)	24921.55(22116.49,27389.41)	2.61(2.43,2.74)	3.28(2.90,3.61)	0.90(0.75,1.06)
Gender					
Male	4016.93(3777.92,4207.07)	13291.89(11907.01,14441.86)	2.97(2.78,3.12)	3.94(3.52,4.29)	1.07(0.91,1.23)
Female	4332.84(3961.41,4568.57)	11629.66(9845.00,13474.35)	2.35(2.14,2.49)	2.78(2.35,3.22)	0.69(0.54,0.84)
SDI					
High SDI	7045.68(6595.37,7331.12)	17654.93(15584.16,19314.09)	6.73(6.29,7.00)	8.58(7.61,9.37)	0.98(0.80,1.17)
High-middle SDI	1018.77(918.24,1126.93)	5056.94(4398.39,5738.21)	1.22(1.09,1.35)	2.77(2.40,3.14)	2.68(2.44,2.92)
Low SDI	1.06(0.43,2.47)	12.19(4.20,24.52)	0.01(0.00,0.01)	0.03(0.01,0.06)	5.88(5.41,6.34)
Low-middle SDI	32.64(26.75,43.33)	333.48(279.58,390.87)	0.07(0.06,0.10)	0.29(0.24,0.34)	4.80(4.69,4.91)
Middle SDI	246.48(198.40,288.50)	1841.82(1590.40,2111.36)	0.31(0.25,0.36)	0.80(0.69,0.91)	3.01(2.88,3.15)
Regions					
Andean Latin America	0.33(0.18,0.61)	72.10(52.34,94.10)	0.02(0.01,0.04)	1.43(1.04,1.87)	14.34(11.63,17.11)
Australasia	240.07(213.85,266.68)	682.40(571.52,790.72)	10.72(9.53,11.91)	12.92(10.84,14.98)	0.69(0.35,1.03)
Caribbean	1.28(1.12,1.56)	114.83(94.23,137.71)	0.06(0.05,0.07)	2.41(1.98,2.89)	10.94(7.47,14.52)
Central Asia	0.38(0.26,0.59)	6.92(5.90,8.08)	0.01(0.01,0.02)	0.11(0.10,0.13)	9.21(8.13,10.31)
Central Europe	101.04(94.94,107.74)	647.55(579.61,725.67)	0.74(0.70,0.79)	2.89(2.59,3.24)	4.72(4.38,5.06)
Central Latin America	54.08(50.99,56.84)	464.14(401.38,531.82)	0.84(0.79,0.88)	2.18(1.88,2.49)	3.08(2.89,3.28)
Central Sub-Saharan Africa	0.14(0.07,0.30)	0.06(0.03,0.24)	0.01(0.00,0.02)	0.00(0.00,0.01)	−5.96(−7.27, −4.63)
East Asia	291.72(172.14,381.62)	1390.32(901.71,1818.74)	0.41(0.25,0.54)	0.67(0.43,0.88)	0.71(0.29,1.14)
Eastern Europe	58.49(41.68,85.02)	1053.05(947.49,1173.92)	0.24(0.17,0.36)	3.11(2.80,3.47)	8.56(7.59,9.53)
Eastern Sub-Saharan Africa	0.26(0.11,0.51)	0.15(0.07,0.48)	0.00(0.00,0.01)	0.00(0.00,0.00)	−5.14(−6.11, −4.16)
High-income Asia Pacific	601.09(561.76,630.52)	2714.45(2296.20,3022.18)	3.40(3.17,3.57)	5.71(4.89,6.32)	1.73(1.60,1.86)
High-income North America	2701.56(2501.94,2824.41)	6482.71(5785.39,6938.02)	7.80(7.22,8.16)	10.13(9.04,10.85)	0.93(0.49,1.37)
North Africa and Middle East	27.21(10.70,64.23)	275.05(163.69,442.00)	0.22(0.08,0.51)	0.79(0.47,1.28)	4.66(4.39,4.93)
Oceania	0.06(0.03,0.09)	0.08(0.04,0.14)	0.03(0.02,0.05)	0.02(0.01,0.03)	−2.79(−3.58, −2.00)
South Asia	9.38(4.13,22.01)	141.09(73.03,212.19)	0.02(0.01,0.05)	0.12(0.06,0.17)	5.35(5.19,5.52)
Southeast Asia	6.79(3.25,10.96)	51.88(34.10,71.26)	0.04(0.02,0.06)	0.10(0.07,0.14)	3.38(3.26,3.50)
Southern Latin America	15.47(14.12,16.85)	353.34(305.86,407.36)	0.37(0.34,0.41)	4.35(3.77,5.02)	6.70(4.97,8.45)
Southern Sub-Saharan Africa	1.45(0.34,2.52)	1.55(0.81,2.78)	0.07(0.02,0.11)	0.03(0.02,0.06)	−2.75(−3.26, −2.24)
Tropical Latin America	105.30(97.37,112.00)	904.17(808.23,989.79)	1.46(1.34,1.55)	4.06(3.62,4.44)	3.77(3.65,3.90)
Western Europe	4133.60(3864.89,4338.53)	9565.57(8235.79,10785.93)	7.36(6.88,7.72)	10.44(9.06,11.75)	1.46(1.31,1.62)
Western Sub-Saharan Africa	0.06(0.03,0.08)	0.15(0.09,0.23)	0.00(0.00,0.00)	0.00(0.00,0.00)	0.64(0.36,0.92)

MND, motor neuron disease; SDI, socio-demographic index; EAPCs, estimated annual percentage changes.

In 2021, the global MND cases of incident and prevalent among elderly adults aged more than 65 years were 27,817 and 87,396, respectively. These figures increased substantially compared with 1990, which indicating that a great number of elderly patients were still experiencing the threat of the MND. Additionally, the ASIR and ASPR had also increased. The ASIR among elderly adults in 2021 was 3.63 [95% uncertainty intervals (UI): 2.95–4.36], higher than the rate in 1990 (3.28, 95% UI: 2.57–4.05), with an EAPC of 0.43 [95% confidence interval (CI): 0.38–0.49]. Similarly, the ASPR among elderly adults in 2021was 11.45 (95% UI: 8.69–14.88), higher than the rate in 1990 (10.43, 95% UI: 7.58–13.86), with an EAPC of 0.58 (95% CI: 0.48–0.68).

The global cases of DALYs for elderly MND increased from 161,323 in 1990 to 463,947 in 2021, and the ASDR increased from 48.53 in 1990 to 59.92 in 2021, with an EAPC of 0.84 (95% CI, 0.71–0.96). Similarly, the global trends in the number of death cases (from 8,350 to 24,922) and ASMR (from 2.61 to 3.28) also showed upward trends during this period, with a corresponding EAPC for ASMR of 0.90 (95%CI: 0.75–1.06).

### 3.2 Global trends by sex

In 2021, the global ASIR of MND was generally higher in older men than in women, with a risk ratio of 1.35 (Supplementary Table S3). The sex ratio (male-to-female) in incident and death cases gradually decreased with age. Conversely, the sex ratio in incidence and mortality gradually increases with age, peaking in the 90–94 years age group (Supplementary Table S3). From 1990 to 2021, the global ASIR, ASPR, ASMR, and ASDR of elderly MND were consistently higher in men than those in women across all age subgroups (Supplementary Figure S1). It is worth noting that the ASIR and ASPR of MND increased significantly more in men than in women, with EAPCs of 0.58 versus 0.25 and 0.73 versus 0.38, respectively. In addition, the mortality rate experienced by men was the highest, with an EAPC of 1.07 (95% CI 0.91–1.23) (Supplementary Table S1).

### 3.3 Global trends by age group

Compared with 1990, global cases of incident, prevalent, death and DALYs in 2021 doubled across various age subgroups within the 65–84 age range (65–69, 70–74, 75–79, and 80–84 years). Notably, in the ≥85 age groups (85–89, 90–94, and ≥95 years), the increase was even more pronounced, with cases more than tripling. In general, more than 50% of the cases were observed in the 65–74 age group. In 2021, the ASIR (4.29, 95% UI: 3.53–4.98), ASPR (13.59, 95% UI: 10.73–17.23) and ASDR (69.41, 95% UI: 62.06–76.14) of elderly MND were relatively highest in the 75-79y age group, before rapidly declining after the age of 80. Conversely, the highest ASMR (4.22, 95% UI: 3.50–4.72) was observed in the 80–84 age group, closely followed by the 75–79 age group (ASMR, 4.19, 95% UI: 3.74–4.60). From 1990 to 2021, all age subgroups showed increases in ASIR, ASPR, ASMR and ASDR. These findings are detailed in Supplementary Table S4.

### 3.4 Global trends by SDI

In 2021, the high SDI region accounted for 67.44, 71.58, 70.91 and 69.33% of the incident, prevalent, death and DALY cases among elderly MND patients, respectively. Notably, high SDI region exhibited the highest ASIR [9.22 (95% UI, 7.62–10.80)], ASPR [30.70 (95% UI, 23.91–38.78)], ASMR [8.58 (95% UI, 7.61–9.37)], and ASDR [159.90 (95% UI, 144.12–173.41)] among SDI groups, while the lowest rates were observed in the low or low-middle SDI regions. From 1990 to 2021, it is worth noting that from 1990 to 2021, the ASIR and ASPR for elderly MND in low SDI region showed a decreasing trend, with EAPCs of −0.40 (95%CI, −0.50, −0.30) and −0.04 (95%CI, −0.12, 0.05), respectively. Whereas ASMR and ASDR demonstrated an upward trend, with EAPCs of 5.88 (95%CI, 5.41–6.34) and 2.48(95%CI, 2.15–2.82), respectively.

### 3.5 Global trends by region

The burden and trends of MND varied significantly across different regions. In 2021, Australasia showed the highest ASIR [4.12(95% UI, 3.95–4.29)], ASMR [3.10 (95% UI, 2.73–3.45)], and ASDR [73.50 (95% UI, 66.28–80.70)]. Meanwhile, Western Europe reported the highest ASPR [15.45(95% UI, 13.31–17.92)]. Overall, the top three regions in terms of these four ASRs were Australasia, High-income North America, and Western Europe. From 1990 to 2021, Tropical Latin America exhibited the most substantial increases in ASIR and ASPR, with EAPCs of 2.36 (95% CI, 2.19–2.52) and 1.81 (95% CI, 1.66–1.97), respectively. In contrast, Andean Latin America exhibited the highest increases in ASMR and ASDR, with EAPCs of 15.68 (95% CI, 14.16–17.23) and 10.51 (95% CI, 9.72–11.31), respectively.

### 3.6 National trends

**Figure 1:**
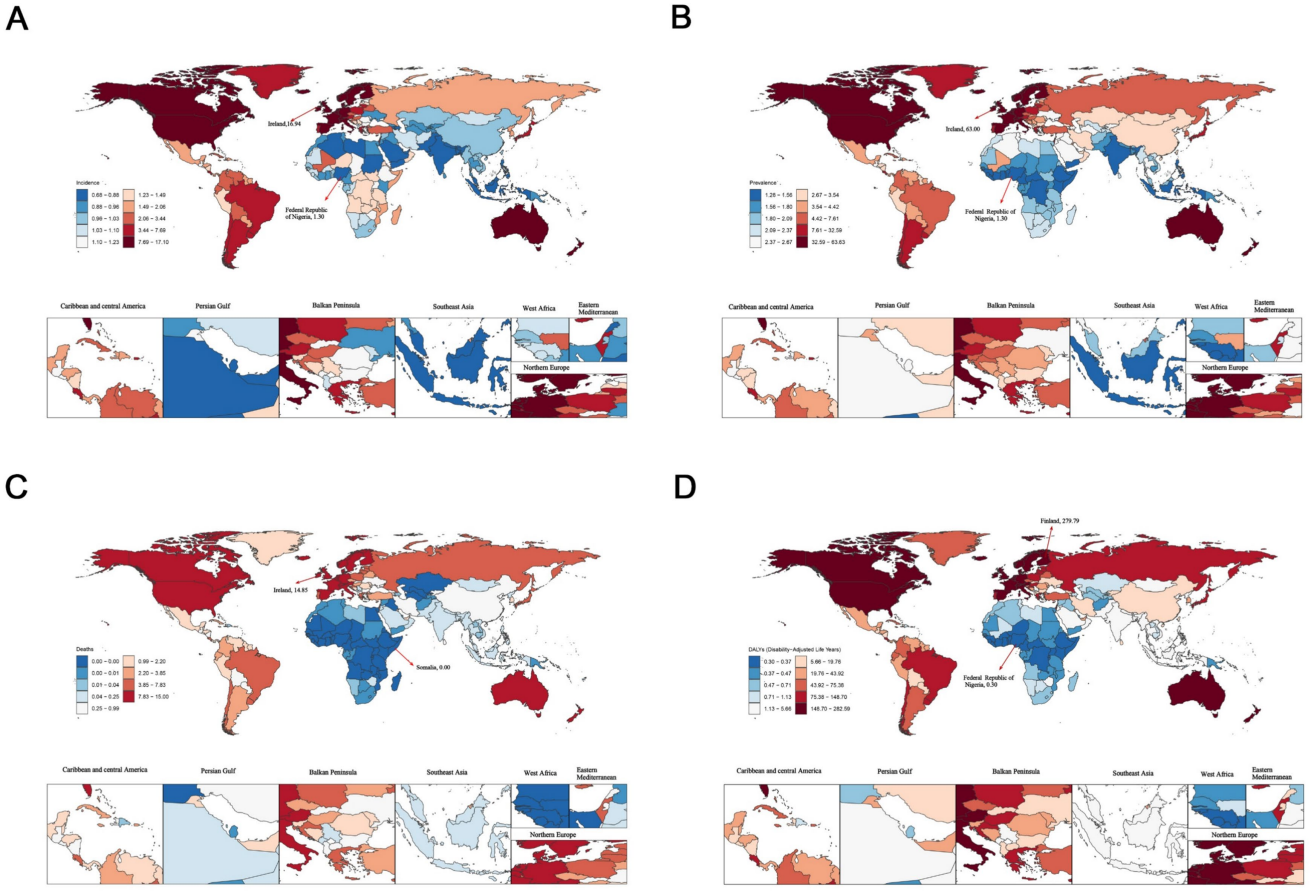
The age-standardized rates of elderly motor neuron disease across 204 countries and territories in 2021. **(A)** Age-standardized incidence rate (ASIR); **(B)** age-standardized prevalence rate (ASPR); **(C)** age-standardized mortality rate (ASMR); **(D)** age-standardized disability-adjusted life years (ASDR).

In 2021, Ireland showed the highest ASIR [16.94; (95%UI, 12.82–20.84)], ASPR [63.00; (95%UI, 44.67–86.65)] and ASMR [14.85; (95%UI, 11.56–18.46)]. Meanwhile, the Republic of Finland recorded the highest ASDR [279.79; (95%UI, 217.83–357.20)]. In contrast, the Federal Republic of Nigeria had the lowest values in ASPR [1.30; (95%UI, 0.69–2.15)] and ASDR [0.30; (95%UI, 0.16–0.51)] (Figure 1 and Supplementary Table S5).

**Figure 2:**
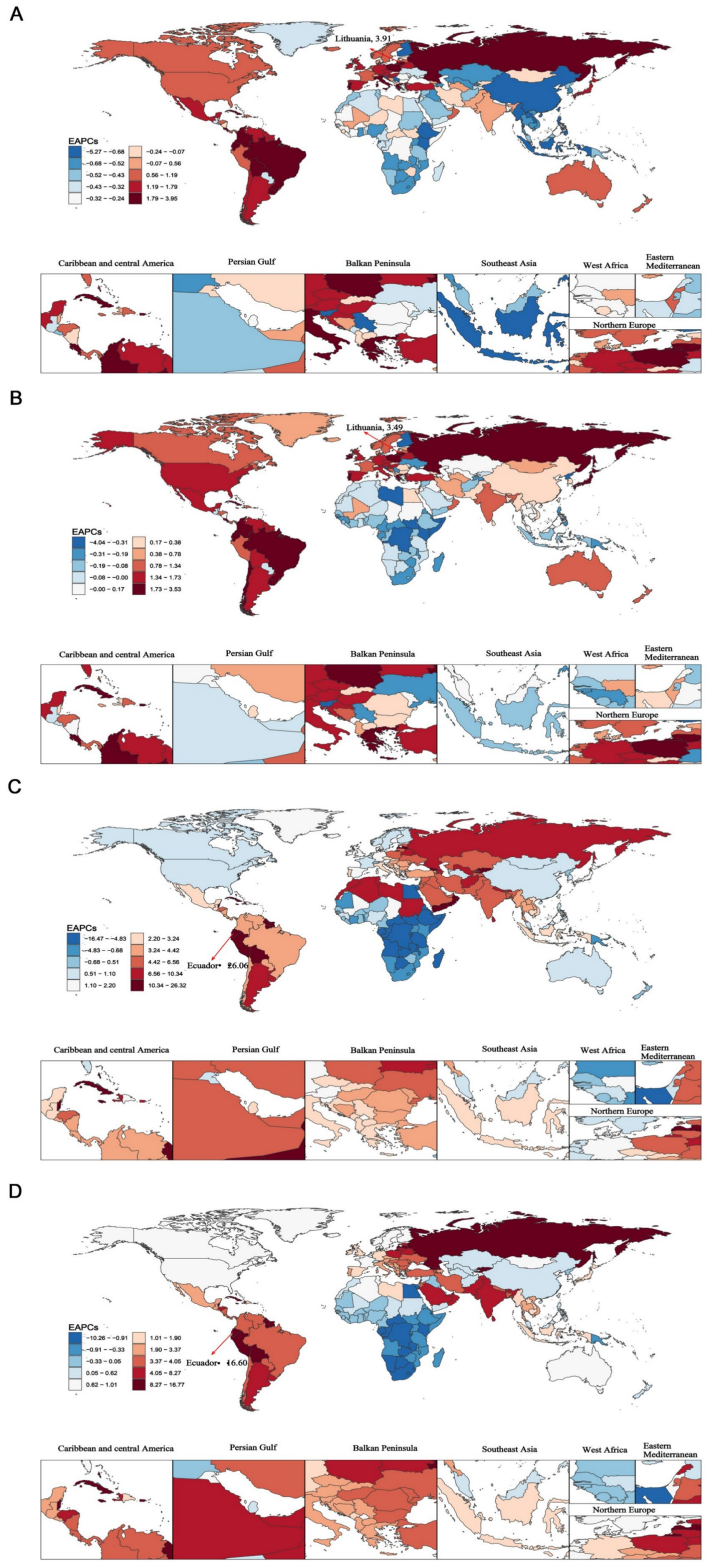
The global estimated annual percentage changes (EAPCs) of elderly motor neuron disease across 204 countries and territories from 1990 to 2021. **(A)** Age-standardized incidence rate (ASIR); **(B)** age-standardized prevalence rate (ASPR); **(C)** age-standardized mortality rate (ASMR); **(D)** age-standardized disability-adjusted life years (ASDR).

From 1990 to 2021, the Republic of Lithuania exhibited the highest increases in ASIR [EAPC: 3.91; 95%CI (3.52–4.31)] and ASPR [EAPC: 3.49; 95%CI (3.24–3.75)]. Conversely, the Republic of Ecuador exhibited the highest increases in ASMR [EAPC: 26.06; 95%CI (18.32–34.30)] and ASDR [EAPC: 16.60; 95%CI (12.61, 20.73)]. Notably, Guam showed the most substantial decreases in ASIR [EAPC: −5.22; 95%CI (−5.89 to −4.54)], ASPR [EAPC: −4.00; 95%CI (−4.52 to −3.48)] and ASDR [EAPC: −10.16; 95%CI (−11.20 to −9.10)] (Figure 2 and Supplementary Table S6).

### 3.7 Trends between SDI and elderly MND

**Figure 3:**
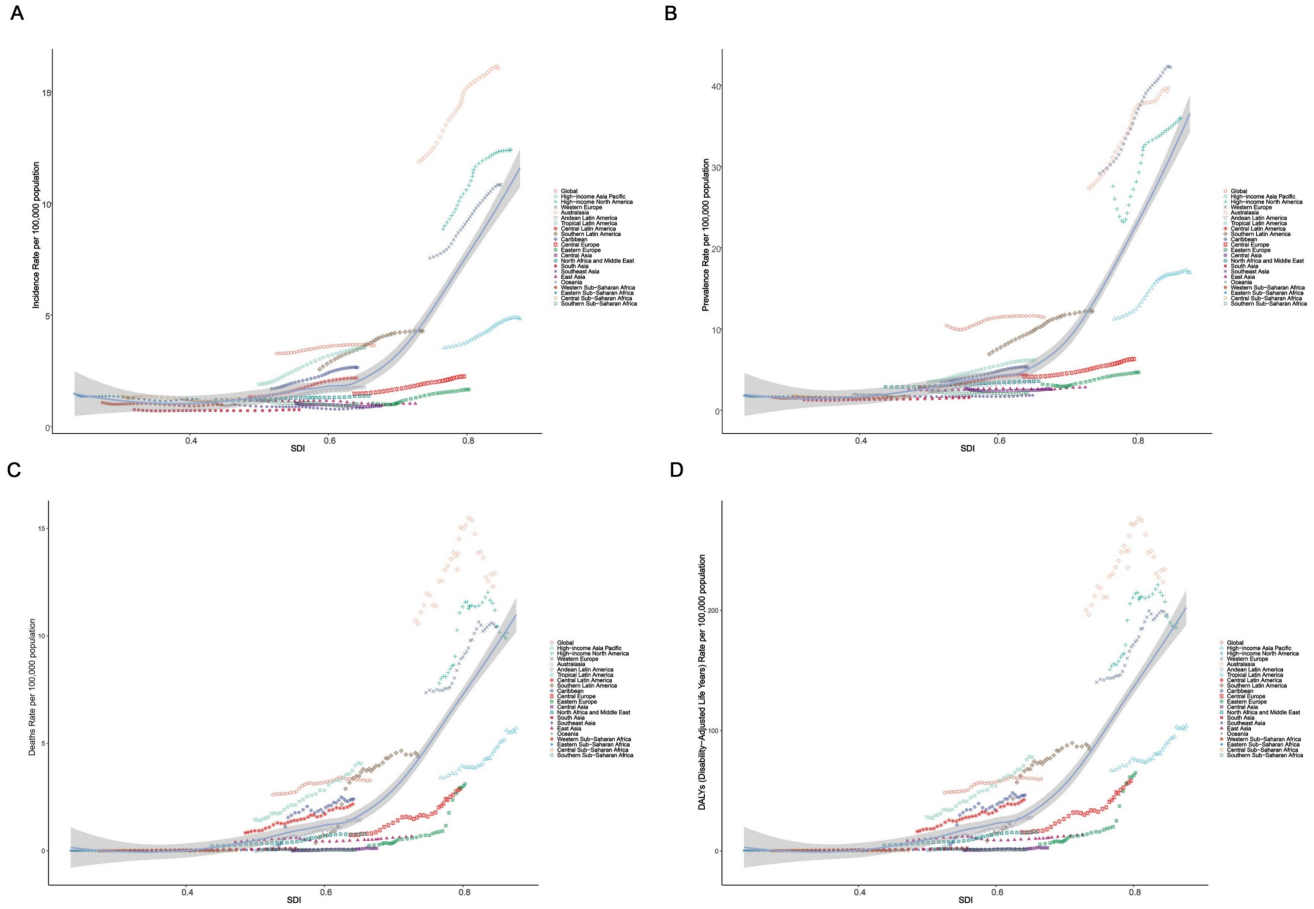
The correlation between socio-demographic index (SDI) and age-standardized rate of elderly motor neuron disease in 2021 among 21 GBD regions. **(A)** Age-standardized incidence rate (ASIR); **(B)** age-standardized prevalence rate (ASPR); **(C)** age-standardized mortality rate (ASMR); **(D)** age-standardized disability-adjusted life years (ASDR).

**Figure 4:**
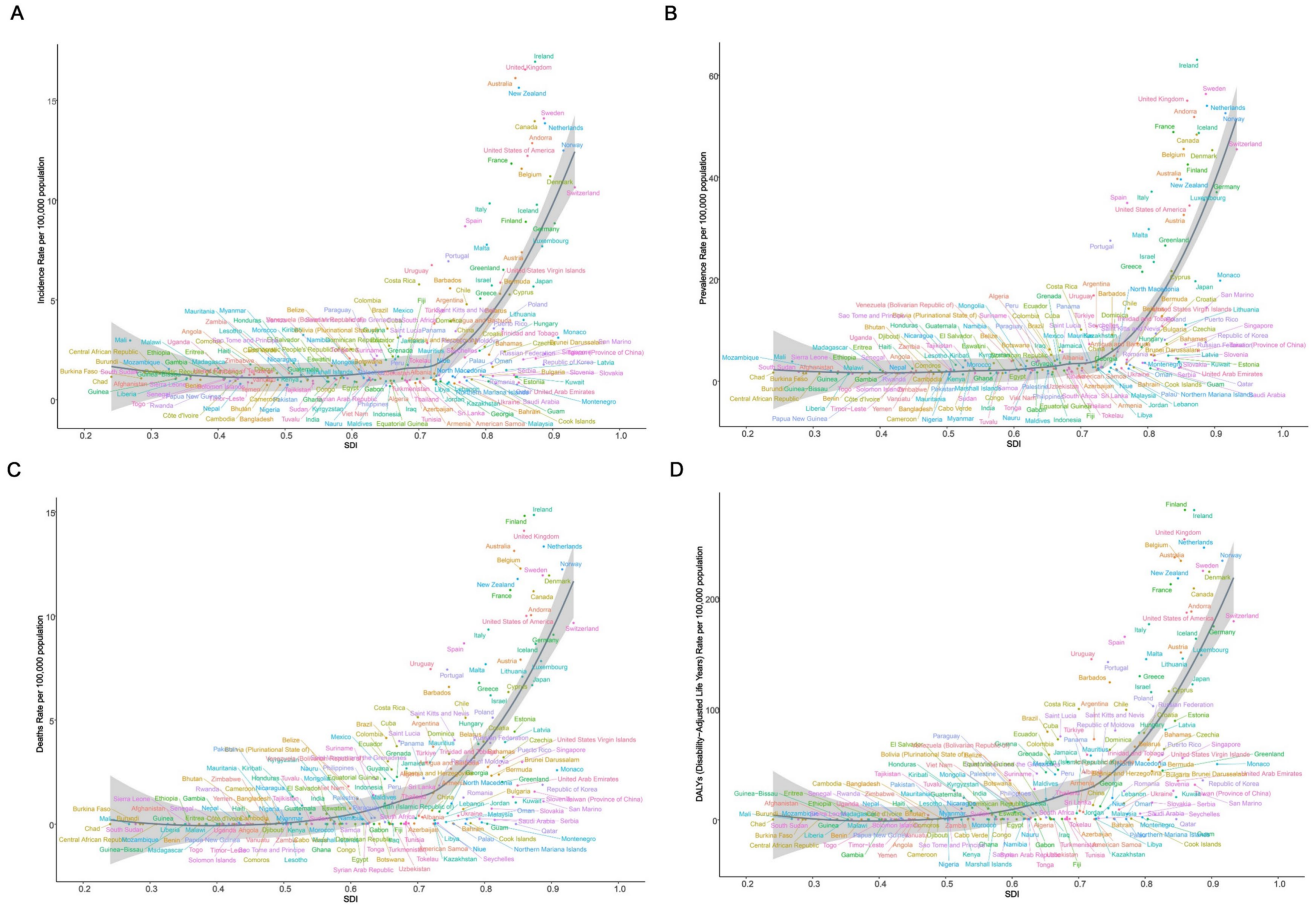
The correlation between socio-demographic index (SDI) and age-standardized rate of elderly motor neuron disease in 2021 across 204 countries and territories. **(A)** Age-standardized incidence rate (ASIR); **(B)** age-standardized prevalence rate (ASPR); **(C)** age-standardized mortality rate (ASMR); **(D)** age-standardized disability-adjusted life years (ASDR).

We analyzed the association of ASIR, ASPR, ASMR, and ASDR across 21 regions and 204 countries to reveal the relationship between the disease burden in elderly MND and the level of social development. At the regional level, Spearman rank order correlation analyses showed significant positive correlation between the SDI and ASIR (*ρ* = 0.63, *p* < 0.0001), ASPR (*ρ* = 0.84, *p* < 0.0001), ASMR (*ρ* = 0.84, *p* < 0.0001), and ASDR (*ρ* = 0.84, *p* < 0.0001). These results suggest that with the increase of SDI level, the burden of elderly MND gradually rises, especially when the SDI exceeds 0.7, at which point the burden of MND increases sharply (Figure 3). Similar significant positive associations were observed at the national level (Figure 4).

### 3.8 Age-period-cohort effects on elderly MND

**Figure 5:**
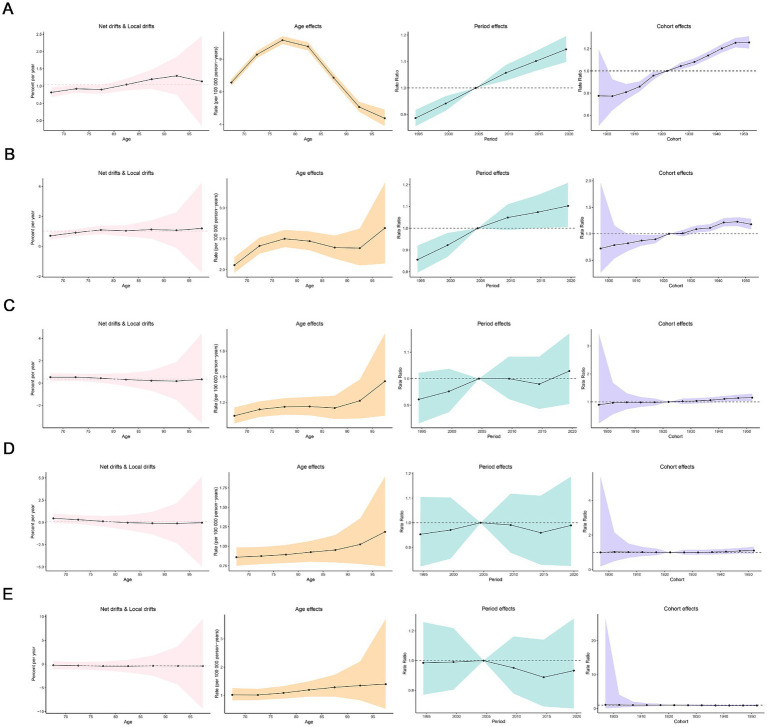
Age, period and cohort effects on elderly motor neuro diseases incidence in socio-demographic index (SDI) regions. **(A)** High SDI region; **(B)** high-middle SDI region; **(C)** middle SDI region; **(D)** low-middle SDI region; **(E)** low SDI region.

Figure 5 showed the incidence of Age-Period-Cohort effects derived from the APC model. A consistent age-related pattern was observed in high-middle, middle, low-middle, and low SDI regions, with ASIR generally increasing with age. However, in high SDI region, the age effects showed a non-linear pattern, initially rising and then declining, with the highest risk observed in individuals aged 75–80 years. Over the period from 1992 to 2021, the period effects have generally exhibited an upward trend in high and high-middle SDI regions, with a significant increase in the period risk observed after 2005. Furthermore, the cohort effects revealed a fluctuating but rising risk of MND incidence generations born after 1922 continue in high and high-middle SDI regions.

### 3.9 Cross-country inequality analysis

**Figure 6:**
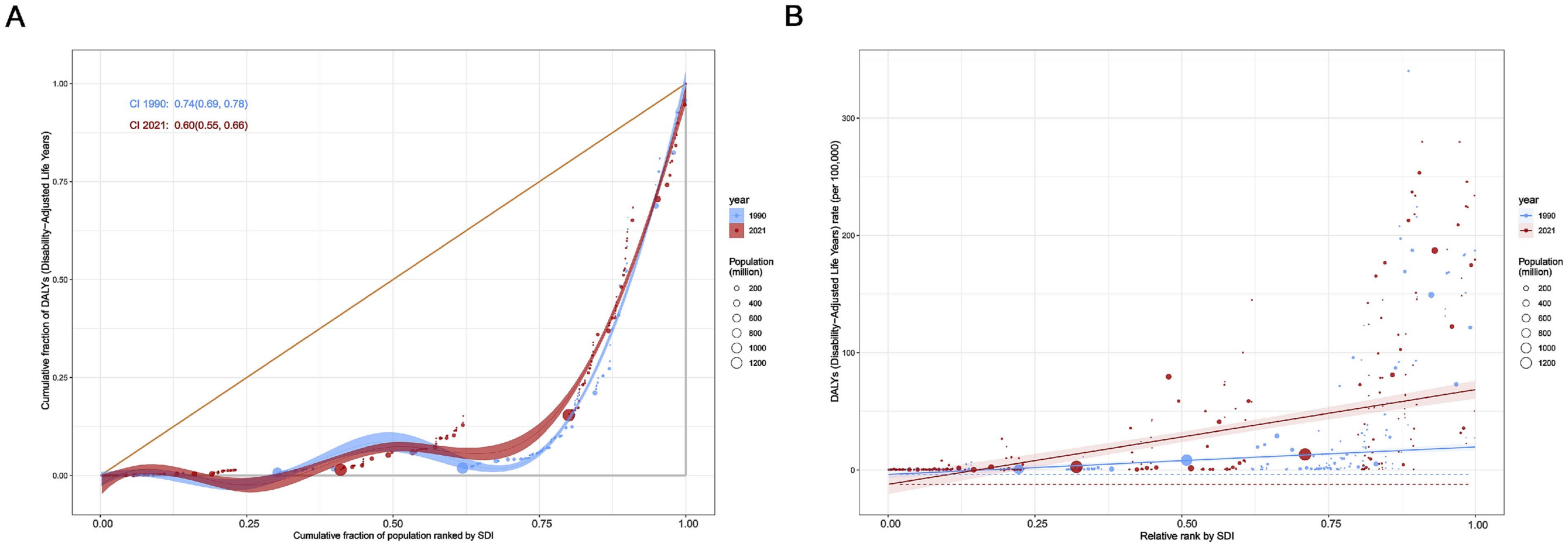
Health inequality regression curves **(A)** and concentration curves **(B)** for global age-standardized disability-adjusted life years (DALYs) rate of elderly motor neuron disease, 1990–2021.

Significant absolute and relative inequalities in the burden of MND were found to be associated with the SDI, with countries and territories with higher SDI values experiencing a higher burden. The inequality slope index indicated that the gap in the DALYs rate between countries and territories with the highest and lowest SDI values increased from 23.46 (95% CI: 18.52–28.40) in 1990 to 80.70 (95% CI: 65.07–96.32) in 2021 (Figure 6A). The Concentration Index was 0.74 (95% CI: 0.69 to 0.78) in 1990 but decreased to 0.60 (95% CI: 0.55 to 0.66) in 2021 (Figure 6B). From 1990 to 2021, while absolute health inequalities in the burden of elderly MND increased, relative inequalities decreased. These findings suggest that relative inequality has been alleviated. Although the ASDR remains high in high SDI countries, the relative gap in burden of MND between countries is gradually narrowing.

### 3.10 Predicted trends

**Figure 7:**
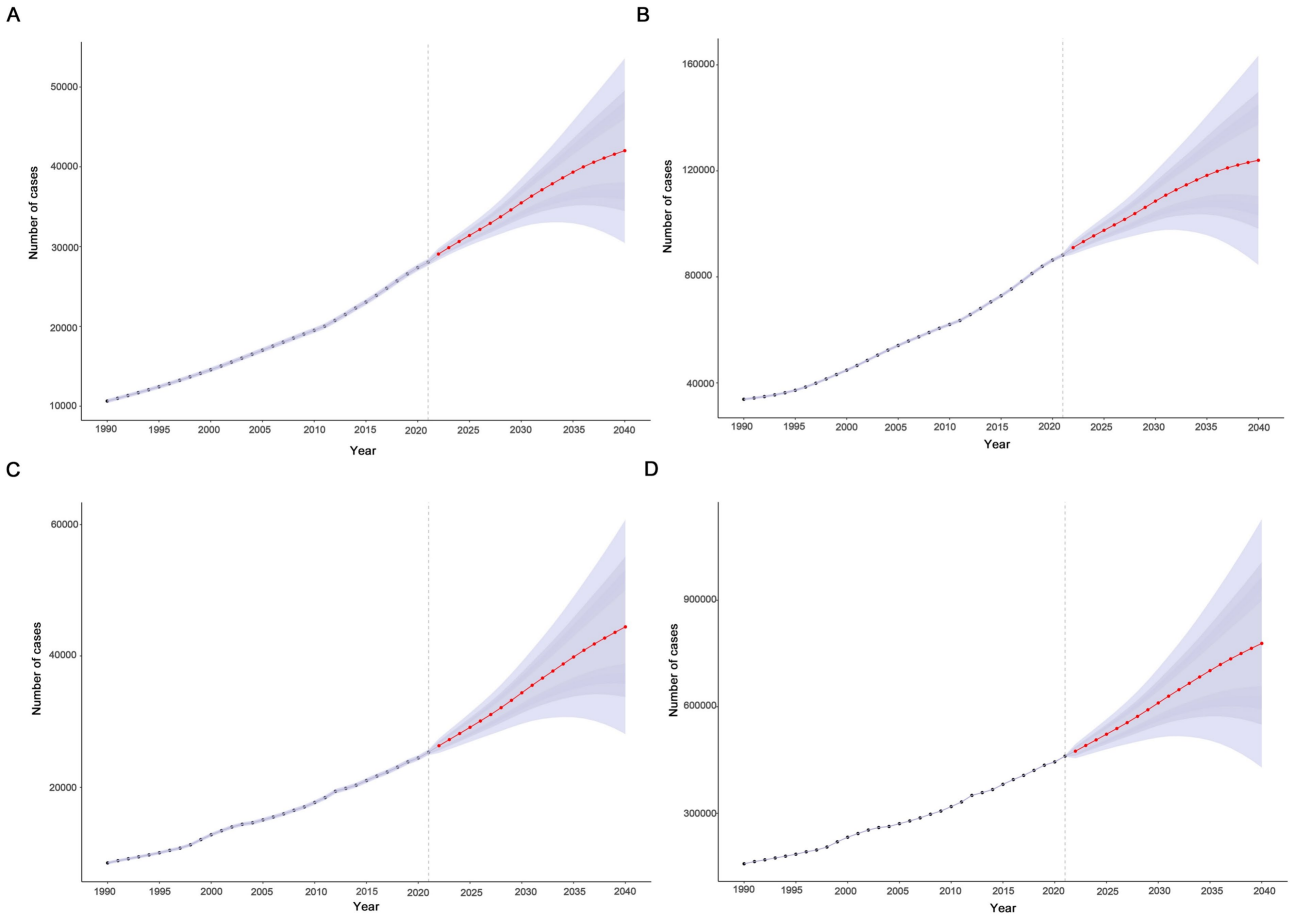
Projected global trends in cases **(A–D)** of elderly motor neuron disease from 1990 to 2040. **(A)** Incident; **(B)** prevalent; **(C)** death; **(D)** disability-adjusted life years. The blue – shaded area represents the 95% uncertainty interval (95% UI).

**Figure 8:**
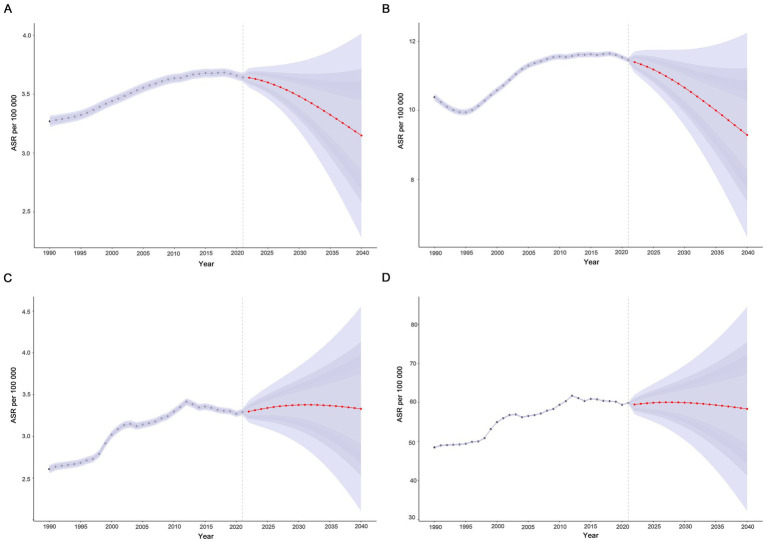
Projected global trends in age-standardized rates **(A–D)** of elderly motor neuron disease from 1990 to 2040. **(A)** Age-standardized incidence rate (ASIR); **(B)** age-standardized prevalence rate (ASPR); **(C)** age-standardized mortality rate (ASMR); **(D)** age-standardized disability-adjusted life years (ASDR). The blue – shaded area represents the 95% uncertainty interval (95% UI).

In this study, the BAPC model was used to predict and analyze the epidemic trend of MND in the elderly over the next 20 years. The results showed that a linear increases in the number of incident, prevalent, death, and DALYs of MND in the elderly patients from 2021 to 2040 (Figures 7A–D and Supplementary Table S7). However, the trends of the four ASRs were different. ASIR and ASPR showed a downward trend, while ASMR and ASDR fluctuated little and tended to be stable. ASIR and ASPR decreased from 3.64 (95%UI: 3.60–3.68) and 11.46 (95%UI: 11.39–11.53) in 2021 to 3.15 (95%UI: 2.28–4.01) and 9.29 (95%UI: 6.33–12.25) in 2040, respectively (Figures 8A,B). Compared with the ASMR (3.29, 95%UI: 3.25–3.33) and ASDR (59.87, 95%UI: 59.69–60.04) values in 2021, the predicted values in 2040 will be 3.32 (95%UI: 2.11–4.55) and 58.32 (95%UI: 32.14–84.51), respectively (Figures 8C,D and Supplementary Table S7). In addition, trends varied across SDI regions, with a decreasing trend in the disease burden projected for high SDI regions, high-middle SDI regions, and middle SDI regions (Supplementary Figures S2A–H, S3). In contrast, the burden of elderly MND was projected to increase in low-middle SDI regions and low SDI regions (Supplementary Figures S4A–H).

## 4 Discussion

Given the rarity of MND, investigating its epidemiology presents significant challenges. Elderly individuals, as a particularly vulnerable population, deserve special attention in the context of MND. To the best of our knowledge, this study provides the first comprehensive analysis of the global burden of MND among elderly patients across various levels, with projections to 2040. Our findings provide valuable insights that can serve as a foundation for public health interventions and clinical practice, ultimately aiming to reduce the global burden of MND in the elderly population.

The findings from our study, which focuses on the elderly population, diverge from those of the previously released GBD 2019 (10) and GBD 2016 studies (19), which pertained to the general population. According to GBD 2019, the incidence of MND within the general population remains relatively stable, with a declining global disease burden rate. However, our study has observed a significant increase in the number and rate of incidence, prevalence, mortality and DALYs due to MND among older individuals from 1990 to 2021, and we project that the global cases will continue to increase though 2040. This increase in incidence is largely attributable to increasing life expectancy and an aging population, as the age of diagnosis has evolved from around 60 years several decades ago to closer to 70 years nowadays (20). It is noteworthy that the mortality has nearly doubled from 0.29 in 1990 to 0.50 in 2021. Despite advances in diagnostic and therapeutic technologies, the rising trend in incidence in older patients is particularly worrying, highlighting the ongoing challenge of the global burden of elderly MND patients. In addition, compared to the GBD 2016 study (19) (the GBD 2019 study did not show the burden of disease in the age subgroups and could not be compared), the age peak of the prevalence of MND was shifted forward in our GBD 2021 study (from 85 to 89 years forward to 75–79 years). It is also a side effect of the improved accessibility of healthcare resources globally, with more patients being diagnosed at an earlier stage of the disease.

Our study found that the burden of disease in elderly MND patients increased with rising SDI levels, highlighting the impact of different SDI regions on disease prevention and management strategies. High SDI region accounted for more than 50% of the elderly MND burden in our study. This regional disparity may be attributed to various factors such as medical resources, disease perception, demographics, and policy interventions. Compared with low SDI region, high SDI region has invested more in screening and treatment of MND. Most current epidemiological studies on MND originate from Europe, North America, and New Zealand, while there is a paucity of epidemiological studies of MND in low-SDI regions such as a large part of Africa (21). Similar geographic differences in MND incidence have been reported in the United States and Europe in terms of socioeconomic status or access to healthcare systems.

In addition, possible influences beyond SDI were also found in our study. For instance, the High-income Asia Pacific region did not exhibit a high burden of disease. Ethnic variability may also play a role (22), with meta-analyses showing higher ALS prevalence in populations of European origin than in East and South Asian populations (9). In low SDI and low to medium SDI areas, older people with MND showed a decreasing trend in ASIR and ASPR and an increasing trend in ASMR and ASDR. This suggests that the situation of elderly MND patients may be more complex and challenging in low SDI and middle SDI regions. On the one hand, life expectancy in this region is shorter than in high SDI regions, resulting in a relatively lower number of MND cases among the elderly compared to high SDI region. However, there is a possibility of underestimation due to limited access to healthcare. On the other hand, poor access to advanced healthcare, such as the relatively low prevalence of non-invasive ventilators and limited clinical use of novel medications (23, 24), may lead to delayed diagnosis and higher mortality.

In order to quantitatively measure health outcome inequality across the socio-economic status gradient, we also conducted a cross-country inequality analysis of DALYs. This analysis revealed that the ASDR of elderly MND increased in absolute inequality between different SDI countries, while relative inequality was alleviated. It suggests that the difference in ASDR between low SDI countries and high SDI countries is widening, but this difference is narrowing relative to their respective overall disease burden levels. The burden of elderly MND is gradually shifting from high SDI countries to low SDI countries, highlighting the pressures on prevention and control faced by low SDI countries. This trend requires global health policies pay more attention to changes in the disease burden of low SDI countries and promote health equality through technology transfer and international cooperation.

It is worth noting that Ecuador exhibited the fastest growth in ASMR and ASDR for elderly MND between 1990 and 2021. Although the ASIR (2.17 cases per 100,000) and ASPR (3.99 cases per 100,000) in Ecuador in 2021 were lower compared to western countries, their growth rates ranked among the top five globally, reflecting an increasing trend in the disease burden among older MND patients. Ecuador is a multi-ethnic country, comprising pre-Columbian indigenous peoples, European settlers, and African slaves (25). Previous epidemiological studies suggest that admixed populations inherited a greater variety of high-risk genes, and different combinations of these risk genes may reduce the overall disease risk (26). However, the continued growth of epidemiological indicators in Ecuador in this study deserves attention, which may be related to the improvement of early diagnosis ability and the acceleration of population aging. A search of the annual cause-of-death registry system of the National Institute of Statistics and Censuses of Ecuador also found a gradual increase in mortality from MND in the general population, with a peak age of death of 55–70 years (27), and an average mortality rate of between 0.2 and 0.25 per 100,000 population from 1990 to 2016, which was much lower than the ASMR level of this study. This may be due to differences in data sources and coverage. Based on the Ecuadorian National Death Registry System, only the cases diagnosed with MND and recorded in the death certificate may be underreported (such as unconfirmed cases, diagnostic errors, or bias in the categorization of causes of death). The GBD study, on the other hand, integrates data from multiple sources (including death registries, epidemiological surveys, hospital records, etc.) and estimates prevalence through a mathematical model, which had a broader coverage and may have included cases that were not diagnosed but met the clinical characteristics of MND. Another hospital-based study in Ecuador also confirmed that the prevalence of degenerative diseases increased from 5.7% in 1990–1994 to 10.2% in 2005–2009 (28). Given the rapid increase in the disease burden of MND in the elderly in Ecuador, combined with the current context of accelerated population aging, overcoming these challenges in countries with relatively scarce healthcare resources may require localized healthcare strategies based on the needs and capacities of specific regions.

Gender differences in MND have garnered significant attention in recent years (29, 30). Our study revealed that the global ASIR of MND was generally higher among older men than women, with a risk ratio of 1.35 times. Previous studies have also demonstrated that men were more prone to developing MND than women, particularly in older age groups (20), with risk ratios ranging from 1.2 to 1.44 (31–33). Gender disparities play an important role in the pathogenesis of MND, potentially linked to hormonal factors (34), genetics, unequal environmental exposures, and differences in disease management (35). Further analyses of different age groups revealed that the sex ratio (male-to-female) for incident and death cases of MND in 2021 progressively decreased with age. This variation in case numbers may be related to the gender structure of the older population, with women generally having a longer life expectancy (32). In contrast, the sex ratio of ASIR and ASMR for MND in our study gradually increased with age, peaking in the 90–94 years age group, which aligns with finding from previous meta-analyses (34). Sexual dimorphism holds prognostic significance, as males typically experience greater weight loss and poorer respiratory function compared to females, leading to a worse prognosis (36, 37). Additionally, some studies have found that familial MND may be more prevalent in males, and female patients carrying mutations in the SOD1 gene may have longer survival than male patients (38). Overall, current research has shown that MND exhibits sexual dimorphism across various dimensions, encompassing the onset of symptoms and their progression at the anatomical, systemic, and sensory levels. Sex-specific differences in the pathophysiology and mechanisms underlying MND have implications for personalized treatment for the disease. More high-quality original research is needed in these areas to address this critical question.

Our study revealed that the ASIR and ASDR for elderly MND patients peak in the 75–79-year age group and decline rapidly after the age of 80. Patients with MND over 80 years old, often referred to as advanced-age patients, may suffer from a ‘survival bias’, whereby only patients with relatively mild disease and a long survival period survive reach this age. Moreover, MND is more challenging to diagnose in advanced-age patients and may be misdiagnosed as other conditions, resulting in some cases not being accurately counted. Additionally, advanced-age patients are less likely to be referred to tertiary care centers due to their frailty being more frequently attributed to aging rather than pathology. Both factors may contribute to the rapid decline in DALYs and incidence among individuals over 80 years old.

Predictions based on the GBD 2021 database using BAPC models showed that the number of incidents, prevalent, deaths and DALYs for elderly MND will continue to rise globally from 2021 to 2040, suggesting that the impact of the MND in the elderly population will continue to expand. However, ASRs showed different trends, with ASIR and ASPR decreased, ASMR and ASDR stabilized, indicating that although the decline in incidence and prevalence, the lethality and disability of the elderly MND are difficult to be significantly improved in the short term. It will still have a serious and sustained negative impact on the health and quality of life of the elderly MND patients, highlighting the urgency and importance of treatment and rehabilitation. Further analysis from the regional dimension, the trends in different SDI regions differ significantly. Disease burden in high SDI, high-middle SDI, and middle SDI regions is expected to show a decreasing trend, which may be attributed to the relatively better healthcare resources and preventive and control measures in these regions. However, the burden of elderly MND in low-middle SDI and low SDI regions is expected to rise, suggesting that global health equity will continue to exist in the next 20 years, and the burden of disease is shifting from high SDI regions to low SDI regions. It is suggested that we need to focus on the investment of medical resources and the optimization of disease prevention and control strategies in these regions to jointly cope with the global health challenge of MND in the elderly.

MND is a fatal neurodegenerative disorder, and elderly patients undoubtedly exacerbate the challenges in its clinical management. Despite extensive research endeavors, current approaches to MND management remain suboptimal, with significant gaps in prevention, screening, diagnosis, and prognostic assessment. The recent recognition of phenotypic heterogeneity may facilitate earlier identification of MND during disease progression by clinicians. And the development of novel diagnostic criteria and the identification of genetic risk factors could expedite the diagnostic workflow (39). Furthermore, certain molecular pathological biomarkers, such as TDP-43 and the 3R/4R tau ratio expressed in plasma extracellular vesicles, have shown utility in MND screening (40). In addition, the clinical management of MND is heavily reliant on the specialized expertise of neurologists. Data from the World Health Organization 2017 report indicate that the median density of adult neurologists in the Americas is 0.70 per 100,000 population. Notably, this figure is even more inadequate in low-income and lower-middle-income countries, at only 0.03 and 0.13 per 100,000 population, respectively. In low-income regions, where the burden of infectious and chronic diseases is substantial, rare diseases such as MND receive disproportionately less governmental attention (41).

Although no definitive preventive strategies for MND, the etiology of MND is multifactorial. Known genetic mutations account for approximately 70% of familial cases and 15% of sporadic cases, with heritability estimates ranging from 8 to 61%. These findings suggest that non-genetic factors also contribute significantly to disease pathogenesis (42). Prior evidence indicates that persistent organic pollutants, occupational exposures, and various other environmental factors influence MND risk and progression (43, 44). It is hypothesized that comprehensive characterization of the MND exposome could enhance the potential for MND prevention by mitigating exposure to high-risk environmental toxicants (45).

To our knowledge, this is the first comprehensive analysis of the global epidemiological trend of MND focusing on older patients. Our study highlights geographic and social differences in incidence, prevalence, mortality and DALYs associated with elderly MND over the past 32 years, and projects changes over the next 20 years using the BAPC model. This analysis contributes to an in-depth understanding of the epidemiology of elderly MND and may guide the development of personalized health policies. Furthermore, we explore cross-country inequalities in the burden of elderly MND, which may broaden the understanding of the distribution differences of MND in the elderly from a national perspective, and facilitate the rational allocation of limited global medical resources to promote global health.

There are several limitations in this study. Firstly, the conceptualization and classification of MND vary across different regions. MND is a broad term that typically encompasses ALS and other related degenerative conditions affecting motor neurons. In the United States, the terms ALS and MND are often used interchangeably, whereas in the United Kingdom, MND are more commonly preferred term to refer to ALS. However, based on the current GBD data, it is challenging to distinguish between each subtype of MND. Secondly, the availability and completeness of data may be insufficient. The GBD database heavily relies on data reported by national health systems, yet the quality of data varies significantly across countries, particularly in low-income countries. Despite the rigorous statistical methods employed in the GBD database, data bias remains unavoidable. Thirdly, diagnosing MND is inherently challenging, especially among elderly patients who may be under-diagnosed. Furthermore, the diagnostic criteria for MNDs have evolved between 1990 and 2021, potentially leading to variations in diagnostic sensitivity. Fourthly, the GBD database focuses on differences between countries or regions in analyzing health inequalities, but it may not offer in-depth analyses of health inequalities across different regions and social groups. Nevertheless, the analyses derived from the GBD 2021 database still representative global epidemiologic data for the current elderly MND population and can help mitigate biases present in individual studies.

## 5 Conclusion

The burden of elderly MND remains a significant concern, as cases and rates have been rising globally from 1990 to 2021, and the global cases are projected to continue to rise through 2040. Overall, the burden of elderly MND is higher in high SDI region and among males. Moreover, cross-country health inequalities persist, with a noticeable shift in the disease burden toward lower SDI regions. Our study provides valuable insights into the burden of MND in older adults, contributing to a deeper understanding of the disease’s epidemiology. These findings underscore the necessity for tailored interventions to address the unique challenges faced by elderly MND patients.

## Data Availability

The datasets presented in this study can be found in online repositories. The names of the repository/repositories and accession number(s) can be found in the article/Supplementary material.
