# Supplementary material for: Global, regional, and national burden of motor neuron disease in adults aged 65 years and older from 1990 to 2021 and forecast to 2040
Source: Front Neurol. 2025 Jul 24;16:1641887. doi: 10.3389/fneur.2025.1641887 (PMC12328143; doi:10.3389/fneur.2025.1641887)
Supplement: Supplementary file 1 [file Supplementary_file_1.docx]

**Supplementary Appendix**

**Global, regional, and national burden of** **motor neuron disease in adults aged 65 years and older from 1990 to 2021 and forecast to 2040**

**Contents**

[Appendix 1. The calculation method of estimated annual percentage change (EAPC) 2](#_Toc27781)

[Appendix 2 The operational details of Bayesian Age-Period-Cohort (BAPC) 3](#_Toc19356)

[Fig S1. Temporal trends in sex-specifc incidence, prevalence, mortality, and DALY rates of motor neuron disease in various age groups, globally, from 1990 to 2021 4](#_Toc15058)

[Fig S2. Future projections of the burden of elderly motor neuron disease in high Socio-Demographic Index (SDI) (A-D) and high-middle SDI (E-H) regions from 1990 to 2040. 5](#_Toc26988)

[Fig S3. Future projections of the burden of elderly Motor Neuron Disease (MND) in Middle Socio-Demographic Index (SDI) region (A-D) from 1990 to 2040. 6](#_Toc17377)

[Fig S4. Future projections of the burden of elderly Motor Neuron Disease (MND) in and Low-middle Socio-Demographic Index (SDI) (A-D) and Low SDI (E-H) regions from 1990 to 2040. 6](#_Toc12524)

[Table S1 Prevalence of MND in patients aged 65 years and older and related EAPCs from 1990 to 2021 7](#_Toc9981)

[Table S2 DALYs of MND in patients aged 65 years and older and related EAPCs from 1990 to 2021 9](#_Toc13793)

[Table S3 The sex ratio(male-to-female) of incidence and mortality in different age groups in 2021 11](#_Toc31707)

[Table S4 Incidence, prevalence, mortality, and DALYs of MND in patients aged 65 years and older and related EAPCs from 1990 to 2021 12](#_Toc16428)

[Table S5 Age-standardized rates of MND in patients aged 65 years and older across 204 countries in 2021 14](#_Toc9709)

[Table S6 Trends in age-standardized rates of EAPCs among older people with MND in 204 countries, 1990-2021 23](#_Toc15420)

[Table S7 Forecasted global cases and age-standardized rates of elderly motor neuron disease from 2021 to 2040 32](#_Toc17285)

Appendix 1. The calculation method of estimated annual percentage change (EAPC)

The calculation method of EAPC was based on the log-linear regression model. The primary idea was to assume a linear relationship between the disease rate after logarithmic transformation (for example, age-standardized death rate) and time (year). The specific calculation formula was:

y=α+βx+ε

EAPC=100×(exp(*β*)−1)

χ-year, y-the national logarithm of rates, α-the intercept, β- the slope, ε- the random error. Here, *β* is the regression coefficient, which represents the slope of the disease rate with time after logarithmic transformation.

If the EAPC and its 95% confidence interval (CI) were greater than or less than zero, the age-standardized rate of determination indicated an increasing or decreasing trend over time. When the 95% CI encompassed zero, the change in age-standardized rate was considered statistically insignificant, implying that there was no statistical difference between the observed trend and change.

**Appendix 2 The operational details of Bayesian Age-Period-Cohort (BAPC)**

We performed a Bayesian Age-Period-Cohort (BAPC) analysis using the integrated nested Laplace approximation (INLA). To ensure smoothing, the BAPC model assumes that the quadratic differences of all effects adhere to independent mean-zero normal distributions. Specifically, the BAPC model assigns prior distribution of the age effect as follows:


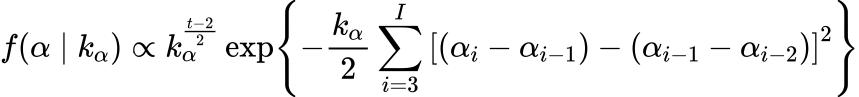


Considering that we are interested in the number of cases in age group *a*, with a *t* period into the future, the following equation can be applied:

**
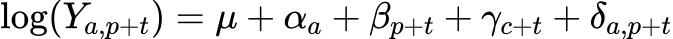
**

Here, we add an independent random effect $\delta_{a,p+t}\sim N(0,k_{\delta}^{-1}$) to adjust for overdispersion. Considering the smoothing assumption, the BAPC models assume prior distribution of the period effect as follows:

**
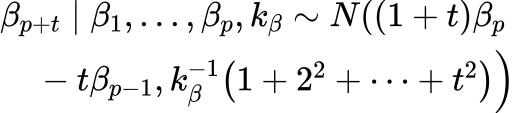
**

We divided the complete dataset into training sets (data between 1990 and 2017) and testing sets (data between 2017 and 2021), which were used to train and validate the predictive models, respectively.

**
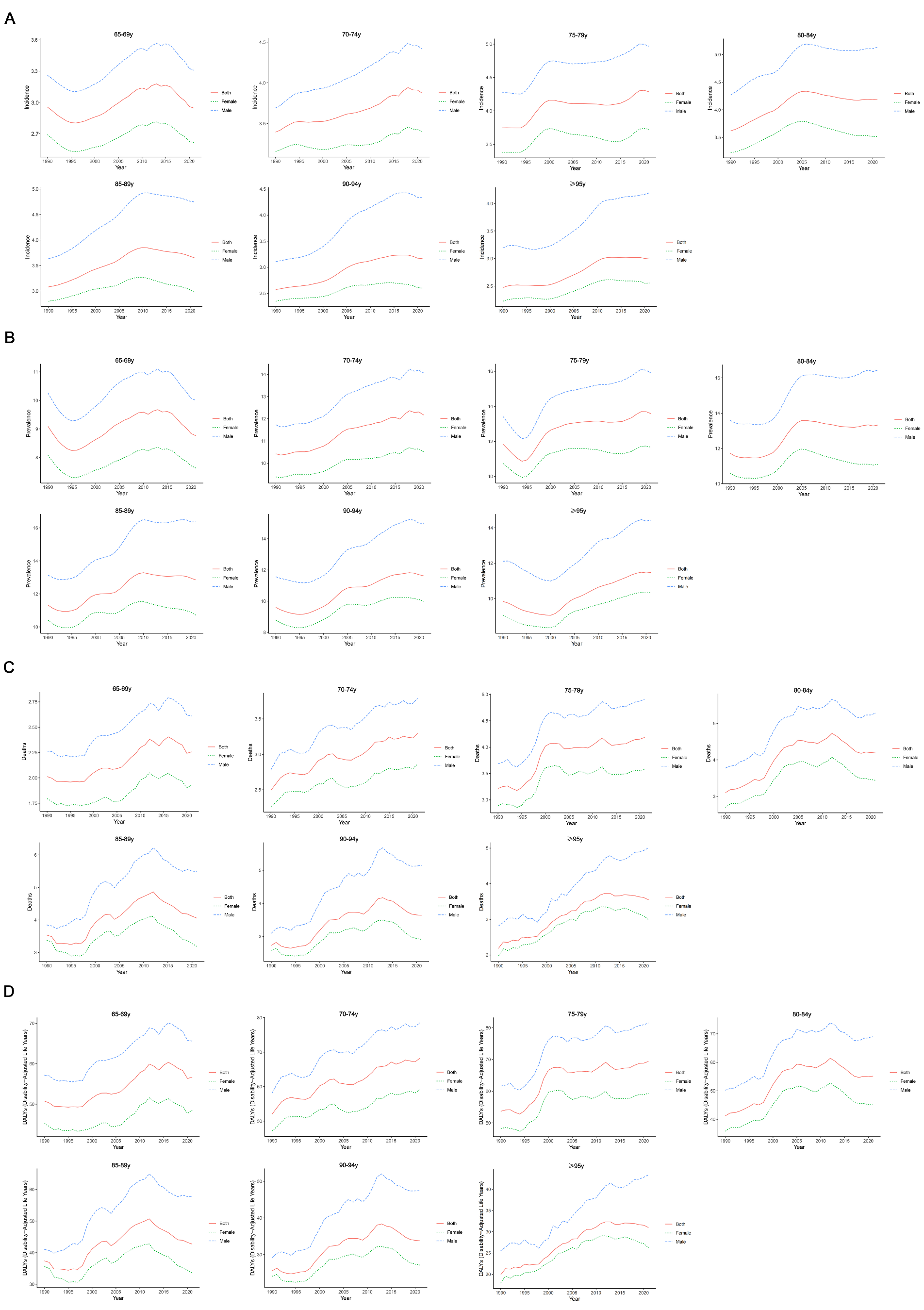
**

Fig S1. Temporal trends in sex-specifc incidence, prevalence, mortality, and DALY rates of motor neuron disease in various age groups, globally, from 1990 to 2021

**
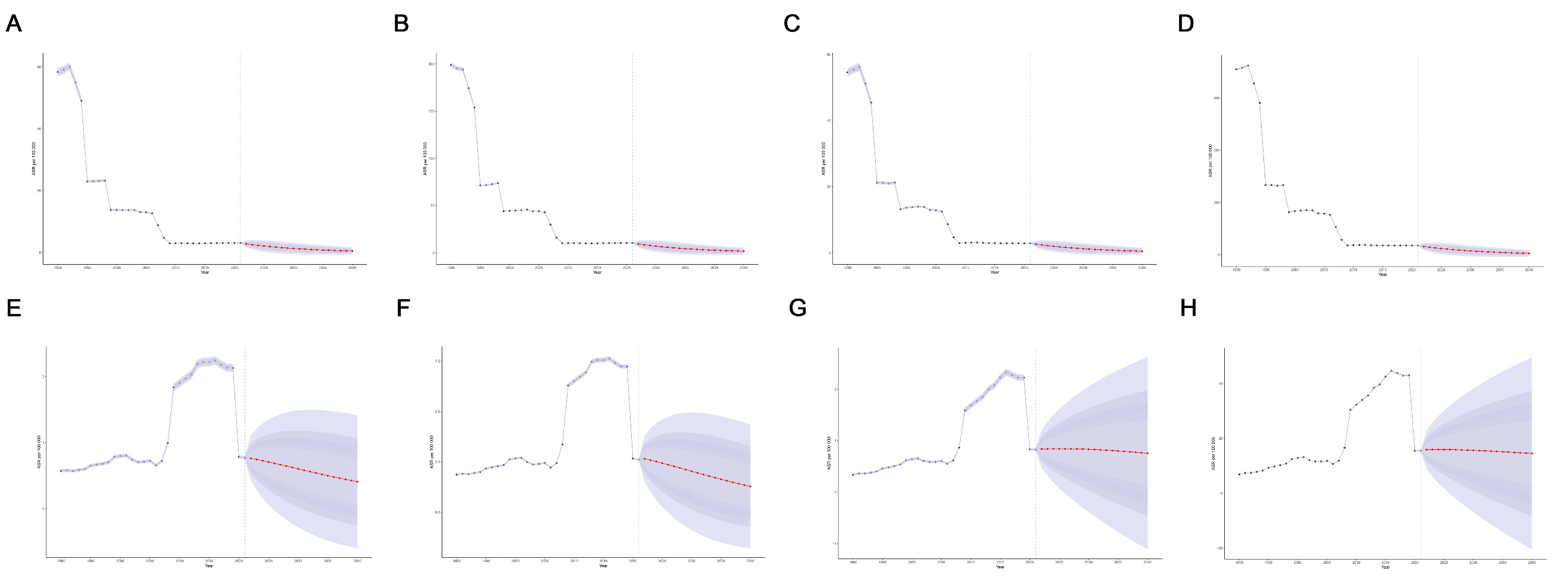
**

# Fig S2. Future projections of the burden of elderly motor neuron disease in high Socio-Demographic Index (SDI) (A-D) and high-middle SDI (E-H) regions from 1990 to 2040.

A/E: Age-Standardized Incidence Rate (ASIR); B/F: Age-Standardized Prevalence Rate (ASPR); C/G: Age-Standardized Mortality Rate (ASMR); D/H: Age-Standardized Disability-Adjusted Life Years (ASDR). The shaded areas represent the uncertainty intervals (95%) around the projections.

**
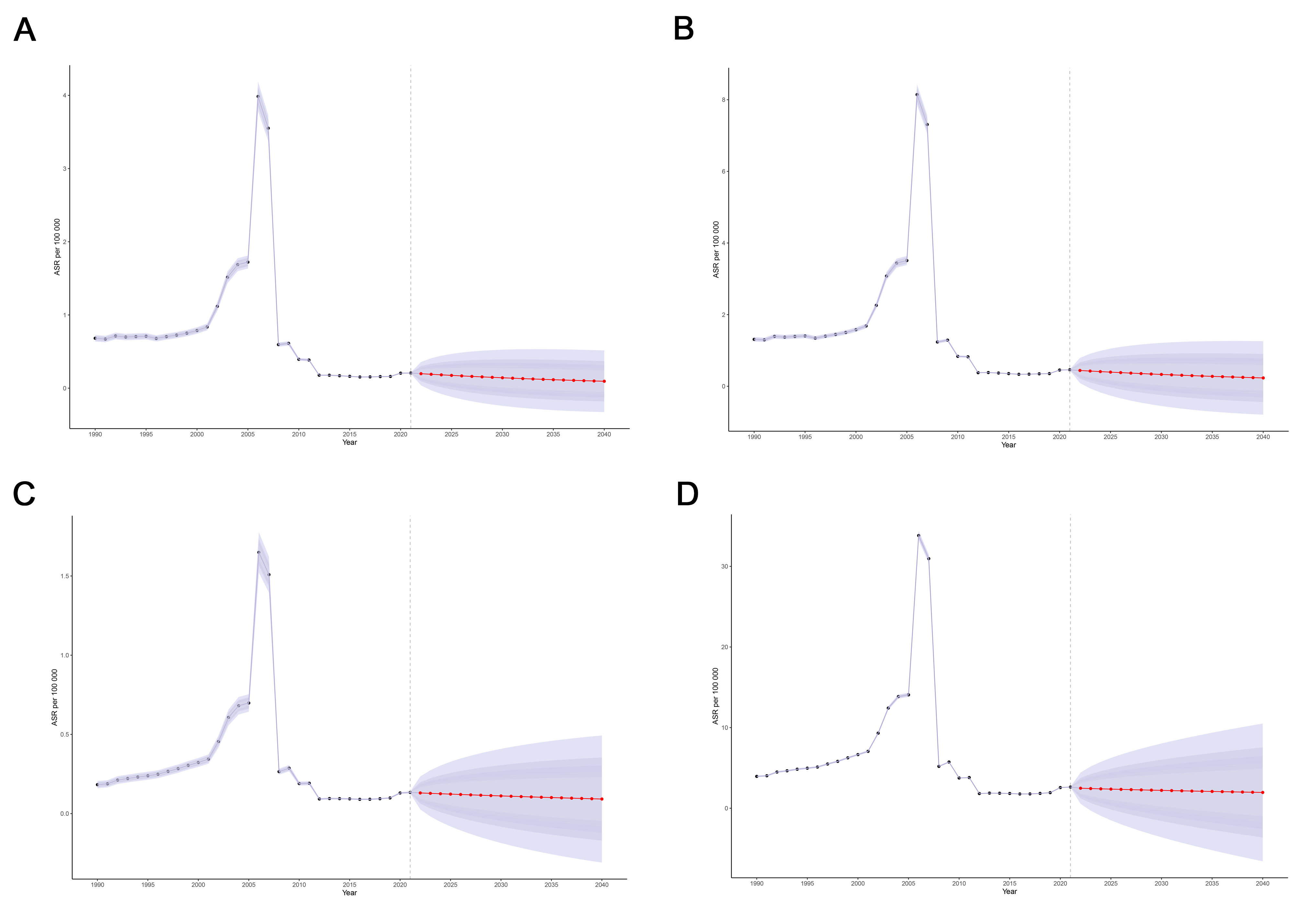
**

# Fig S3. Future projections of the burden of elderly Motor Neuron Disease (MND) in Middle Socio-Demographic Index (SDI) region (A-D) from 1990 to 2040.

A: Age-Standardized Incidence Rate (ASIR); B: Age-Standardized Prevalence Rate (ASPR); C: Age-Standardized Mortality Rate (ASMR); D: Age-Standardized Disability-Adjusted Life Years (ASDR). The shaded areas represent the uncertainty intervals (95%) around the projections.

**
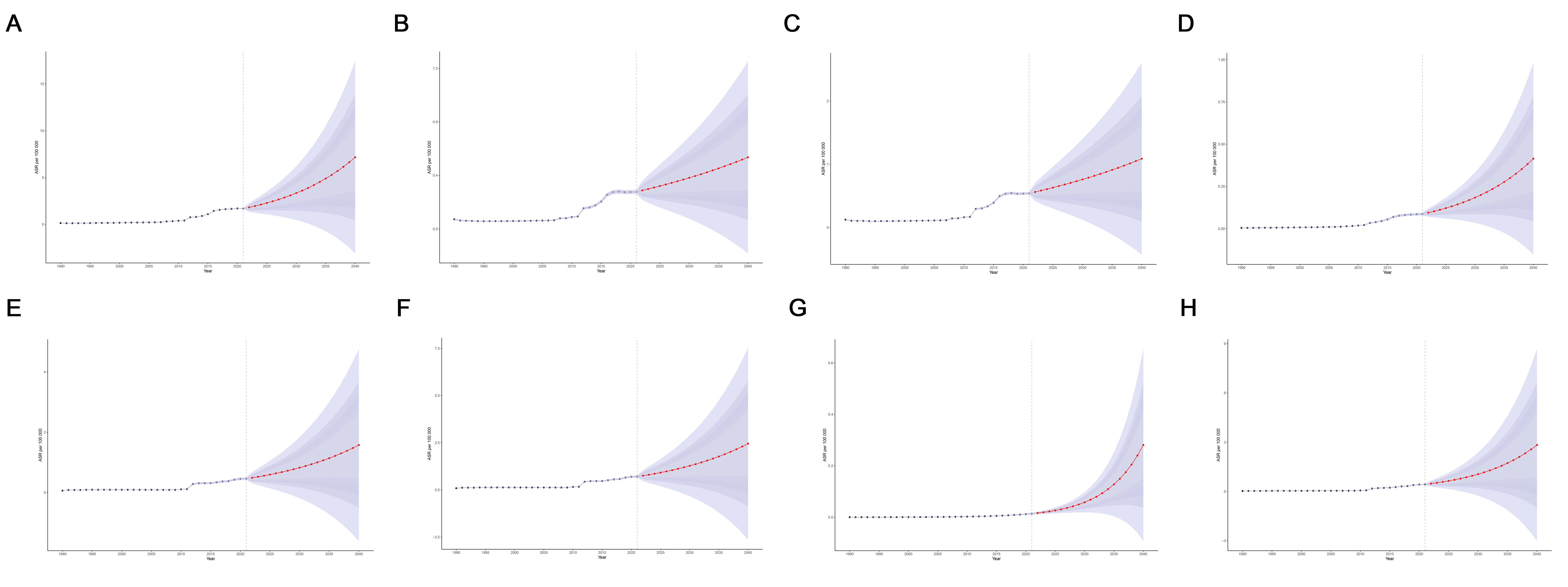
**

# Fig S4. Future projections of the burden of elderly Motor Neuron Disease (MND) in and Low-middle Socio-Demographic Index (SDI) (A-D) and Low SDI (E-H) regions from 1990 to 2040.

A/E: Age-Standardized Incidence Rate (ASIR); B/F: Age-Standardized Prevalence Rate (ASPR); C/G: Age-Standardized Mortality Rate (ASMR); D/H: Age-Standardized Disability-Adjusted Life Years (ASDR). The shaded areas represent the uncertainty intervals (95%) around the projections.

Table S1 Prevalence of MND in patients aged 65 years and older and related EAPCs from 1990 to 2021

| **Characteristics** | **Cases** | | **Age-standardized prevalence rate (per 100,000)** | | **EAPCs** |
| --- | --- | --- | --- | --- | --- |
|  | **1990** | **2021** | **1990** | **2021** | 1990-2021 |
| Global | 33729.97(24622.45,44722.55) | 87395.54(66435.36,113587.52) | 10.43(7.58,13.86) | 11.45(8.69,14.88) | 0.58(0.48,0.68) |
| **Gender** | | | | | |
| Male | 16332.90(12090.70,21550.81) | 46190.27(35348.87,59515.51) | 11.87(8.72,15.72) | 13.51(10.30,17.44) | 0.73(0.62,0.84) |
| Female | 17397.07(12511.41,23292.38) | 41205.27(31021.45,53891.37) | 9.39(6.73,12.60) | 9.82(7.39,12.83) | 0.38(0.29,0.47) |
| **SDI** | | | | | |
| High SDI | 25396.36(18850.14,33216.62) | 62509.82(48575.91,79060.85) | 24.26(17.97,31.76) | 30.70(23.91,38.78) | 1.09(0.96,1.21) |
| High-middle SDI | 5656.25(3882.16,7782.68) | 15922.45(11463.53,21531.88) | 6.88(4.69,9.51) | 8.74(6.29,11.82) | 0.96(0.81,1.11) |
| Low SDI | 247.58(135.68,406.31) | 553.57(311.39,901.81) | 1.60(0.87,2.64) | 1.56(0.87,2.55) | -0.04(-0.12,0.05) |
| Low-middle SDI | 692.74(386.14,1135.79) | 2069.40(1215.49,3241.56) | 1.62(0.89,2.64) | 1.85(1.08,2.90) | 0.57(0.49,0.64) |
| Middle SDI | 1711.25(976.40,2746.62) | 6274.68(3871.90,9462.47) | 2.26(1.27,3.62) | 2.77(1.70,4.17) | 0.83(0.76,0.90) |
| **Regions** | | | | | |
| Andean Latin America | 38.83(22.63,61.42) | 175.75(113.09,257.02) | 2.42(1.41,3.83) | 3.49(2.25,5.11) | 1.53(1.43,1.62) |
| Australasia | 611.14(430.53,829.82) | 2077.70(1466.99,2826.08) | 27.30(19.20,37.12) | 39.66(28.04,53.85) | 1.29(1.18,1.41) |
| Caribbean | 79.46(47.75,122.02) | 254.54(168.83,359.42) | 3.53(2.12,5.43) | 5.34(3.54,7.53) | 1.56(1.47,1.66) |
| Central Asia | 91.71(51.55,147.24) | 148.58(84.52,236.01) | 2.70(1.52,4.33) | 2.62(1.48,4.15) | 0.08(-0.08,0.24) |
| Central Europe | 528.91(320.16,801.09) | 1406.92(928.35,1986.53) | 4.14(2.49,6.28) | 6.31(4.16,8.91) | 1.60(1.53,1.67) |
| Central Latin America | 179.98(107.18,277.87) | 931.15(616.85,1338.24) | 2.81(1.66,4.33) | 4.36(2.89,6.27) | 1.73(1.64,1.81) |
| Central Sub-Saharan Africa | 24.62(13.30,41.13) | 52.72(28.53,87.53) | 1.78(0.96,2.98) | 1.62(0.87,2.70) | -0.26(-0.35, -0.17) |
| East Asia | 1668.31(955.79,2678.10) | 5605.08(3274.07,8696.18) | 2.51(1.42,4.03) | 2.83(1.64,4.39) | 0.33(0.24,0.43) |
| Eastern Europe | 705.91(399.05,1113.51) | 1555.88(989.56,2253.96) | 3.21(1.81,5.06) | 4.65(2.95,6.74) | 1.80(1.57,2.04) |
| Eastern Sub-Saharan Africa | 91.10(48.29,152.09) | 186.95(99.85,311.56) | 1.81(0.96,3.02) | 1.67(0.89,2.79) | -0.26(-0.36, -0.16) |
| High-income Asia Pacific | 1986.67(1373.62,2726.07) | 7660.36(5479.30,10225.78) | 11.24(7.75,15.45) | 16.96(12.18,22.59) | 1.49(1.27,1.71) |
| High-income North America | 9523.38(7000.60,12579.45) | 23068.78(18695.53,28186.91) | 27.57(20.25,36.44) | 35.96(29.16,43.91) | 1.51(1.28,1.75) |
| North Africa and Middle East | 354.77(213.34,545.81) | 1221.48(788.65,1788.61) | 2.89(1.72,4.46) | 3.60(2.31,5.28) | 0.86(0.82,0.91) |
| Oceania | 3.86(2.27,6.17) | 8.46(4.81,13.71) | 1.99(1.15,3.19) | 1.78(1.00,2.88) | -0.54(-0.64, -0.43) |
| South Asia | 498.32(270.68,829.99) | 1818.63(1032.20,2888.32) | 1.28(0.69,2.14) | 1.56(0.88,2.48) | 0.74(0.65,0.83) |
| Southeast Asia | 319.01(174.62,525.87) | 916.33(524.02,1473.80) | 1.76(0.96,2.91) | 1.84(1.04,2.96) | 0.13(0.05,0.22) |
| Southern Latin America | 282.96(179.08,417.66) | 985.81(682.34,1360.74) | 6.91(4.35,10.22) | 12.15(8.41,16.77) | 1.81(1.66,1.97) |
| Southern Sub-Saharan Africa | 46.36(25.16,76.30) | 90.17(49.55,147.40) | 2.31(1.24,3.80) | 2.15(1.17,3.53) | -0.19(-0.30, -0.08) |
| Tropical Latin America | 250.83(155.80,377.78) | 1375.85(911.86,1945.65) | 3.52(2.17,5.31) | 6.18(4.09,8.74) | 2.14(2.01,2.27) |
| Western Europe | 16341.07(12139.64,21239.00) | 37649.22(28301.79,48929.29) | 29.16(21.62,37.93) | 42.22(31.84,54.77) | 1.31(1.22,1.40) |
| Western Sub-Saharan Africa | 102.80(57.02,168.01) | 205.16(115.62,332.50) | 1.60(0.88,2.63) | 1.56(0.87,2.53) | 0.00(-0.08,0.08) |

MND, motor neuron disease; SDI, socio-demographic index; EAPCs, estimated annual percentage changes.

Table S2 DALYs of MND in patients aged 65 years and older and related EAPCs from 1990 to 2021

| **Characteristics** | **Cases** | | **Age-standardized DALYs rate (per 100,000)** | | **EAPCs** |
| --- | --- | --- | --- | --- | --- |
|  | **1990** | **2021** | **1990** | **2021** | 1990-2021 |
| Global | 161323.50(152468.51,168548.99) | 463947.01(418757.82,506931.62) | 48.53(45.68,50.79) | 59.92(53.94,65.53) | 0.84(0.71,0.96) |
| **Gender** | | | | | |
| Male | 79351.06(74868.94,83181.00) | 249634.97(225706.01,270232.30) | 55.18(51.93,57.89) | 70.97(64.00,76.91) | 0.98(0.85,1.11) |
| Female | 81972.44(76317.51,86134.85) | 214312.04(186249.79,245782.23) | 43.58(40.46,45.86) | 50.97(44.27,58.46) | 0.64(0.53,0.75) |
| **SDI** | | | | | |
| High SDI | 134167.84(126955.42,139239.77) | 321367.53(288635.96,349034.60) | 128.21(121.31,133.08) | 159.90(144.12,173.41) | 0.91(0.75,1.07) |
| High-middle SDI | 20854.49(18836.69,23020.96) | 97603.92(85981.58,110539.59) | 24.22(21.85,26.74) | 52.49(46.18,59.47) | 2.52(2.32,2.71) |
| Low SDI | 74.92(42.44,126.80) | 365.28(183.90,624.73) | 0.46(0.26,0.78) | 0.95(0.48,1.62) | 2.48(2.15,2.82) |
| Low-middle SDI | 789.60(644.79,1035.62) | 6940.24(5867.88,8090.85) | 1.71(1.39,2.24) | 5.83(4.92,6.80) | 4.28(4.20,4.35) |
| Middle SDI | 5334.61(4320.95,6260.17) | 37244.07(32341.75,42623.11) | 6.41(5.22,7.51) | 15.56(13.51,17.79) | 2.80(2.67,2.93) |
| **Regions** | | | | | |
| Andean Latin America | 14.67(9.30,22.60) | 1407.15(1027.10,1815.19) | 0.90(0.57,1.39) | 27.80(20.31,35.87) | 12.03(10.03,14.07) |
| Australasia | 4549.30(4068.88,5041.64) | 12125.18(10315.72,13982.48) | 200.30(178.95,222.05) | 234.10(199.45,270.07) | 0.58(0.27,0.90) |
| Caribbean | 41.26(32.73,53.78) | 2204.49(1818.72,2634.43) | 1.80(1.43,2.36) | 46.53(38.39,55.60) | 9.68(6.77,12.66) |
| Central Asia | 27.03(17.29,41.24) | 166.62(141.00,197.98) | 0.79(0.50,1.20) | 2.65(2.23,3.18) | 4.98(4.28,5.68) |
| Central Europe | 2161.24(2027.08,2316.59) | 13107.01(11755.33,14630.62) | 15.63(14.65,16.79) | 58.46(52.43,65.26) | 4.57(4.25,4.90) |
| Central Latin America | 1079.10(1018.66,1138.43) | 9212.21(8005.15,10542.25) | 16.31(15.37,17.22) | 42.65(37.07,48.79) | 3.06(2.86,3.27) |
| Central Sub-Saharan Africa | 8.39(5.05,13.64) | 12.51(6.81,21.80) | 0.55(0.32,0.90) | 0.38(0.20,0.66) | -1.31(-1.59, -1.03) |
| East Asia | 6388.16(3873.78,8271.06) | 29055.68(19348.78,37696.25) | 8.59(5.27,11.12) | 13.56(9.03,17.59) | 0.64(0.22,1.06) |
| Eastern Europe | 1356.38(971.64,1927.29) | 22316.28(20182.72,24750.15) | 5.72(4.09,8.13) | 64.96(58.72,72.16) | 8.20(7.28,9.14) |
| Eastern Sub-Saharan Africa | 25.01(14.43,41.40) | 42.55(22.44,73.52) | 0.47(0.27,0.79) | 0.38(0.20,0.65) | -0.85(-1.01, -0.69) |
| High-income Asia Pacific | 11971.83(11286.33,12541.91) | 46679.66(40511.26,51458.38) | 66.97(63.05,70.20) | 103.77(90.84,113.93) | 1.45(1.32,1.59) |
| High-income North America | 52054.90(48744.34,54350.55) | 121285.51(110279.65,128914.92) | 150.84(141.28,157.49) | 189.53(172.26,201.44) | 0.83(0.43,1.23) |
| North Africa and Middle East | 621.84(292.90,1358.43) | 5717.07(3509.19,8998.48) | 4.73(2.22,10.29) | 15.87(9.76,25.02) | 4.28(4.03,4.53) |
| Oceania | 2.00(1.34,2.93) | 3.41(2.12,5.19) | 1.00(0.64,1.47) | 0.69(0.42,1.06) | -1.67(-2.06, -1.28) |
| South Asia | 301.06(164.11,573.94) | 3237.22(1889.89,4663.30) | 0.71(0.39,1.34) | 2.57(1.50,3.70) | 4.18(3.99,4.38) |
| Southeast Asia | 204.74(126.31,309.56) | 1221.61(860.96,1631.36) | 1.06(0.66,1.61) | 2.29(1.62,3.06) | 2.53(2.46,2.61) |
| Southern Latin America | 358.47(320.82,399.14) | 6851.78(5980.73,7847.89) | 8.52(7.60,9.51) | 84.97(74.18,97.30) | 6.33(4.71,7.97) |
| Southern Sub-Saharan Africa | 40.03(14.96,65.50) | 51.95(31.65,83.98) | 1.80(0.69,2.95) | 1.13(0.68,1.83) | -1.98(-2.38, -1.58) |
| Tropical Latin America | 2126.13(1975.88,2253.49) | 17609.60(15896.63,19220.27) | 28.35(26.28,30.09) | 77.96(70.29,85.10) | 3.65(3.53,3.77) |
| Western Europe | 77969.13(73670.20,81494.04) | 171593.52(150601.09,192355.02) | 140.25(132.61,146.54) | 196.54(173.55,220.07) | 1.37(1.23,1.51) |
| Western Sub-Saharan Africa | 22.83(12.26,38.30) | 45.99(25.32,76.09) | 0.36(0.19,0.60) | 0.35(0.19,0.58) | 0.02(-0.06,0.11) |

MND, motor neuron disease; SDI, socio-demographic index; EAPCs, estimated annual percentage changes.

Table S3 The sex ratio(male-to-female) of incidence and mortality in different age groups in 2021

| **Age group** | **Case** | | **Rate** | |
| --- | --- | --- | --- | --- |
|  | **Incident** | **Death** | **Age-standardized incidence rate (per 100,000)** | **Age-standardized mortality rate (per 100,000)** |
| Global | 1.11(1.09-1.12) | 1.14(1.07-1.21) | 1.35(1.33-1.37) | 1.42(1.33-1.50) |
| 65-69 years | 1.16(1.14-1.18) | 1.24(1.18-1.24) | 1.27(1.24-1.29) | 1.35(1.28-1.36) |
| 70-74 years | 1.14(1.13-1.14) | 1.17(1.10-1.20) | 1.3(1.28-1.30) | 1.32(1.25-1.37) |
| 75-79 years | 1.11(1.10-1.12) | 1.14(1.07-1.20) | 1.33(1.32-1.35) | 1.37(1.29-1.45) |
| 80-84 years | 1.05(1.03-1.08) | 1.1(1.02-1.25) | 1.46(1.43-1.50) | 1.53(1.42-1.74) |
| 85-89 years | 0.96(0.96-0.99) | 1.05(0.96-1.20) | 1.59(1.58-1.64) | 1.72(1.59-1.99) |
| 90-94 years | 0.81(0.77-0.84) | 0.85(0.78-0.96) | 1.67(1.59-1.74) | 1.77(1.62-1.98) |
| 95+ years | 0.63(0.59-0.69) | 0.64(0.60-0.71) | 1.64(1.54-1.80) | 1.68(1.55-1.85) |

Table S4 Incidence, prevalence, mortality, and DALYs of MND in patients aged 65 years and older and related EAPCs from 1990 to 2021

| **Age groups** | **Case** | | **Age-standardized rate (per 100,000)** | | **EAPCs** |
| --- | --- | --- | --- | --- | --- |
|  | **1990** | **2021** | **1990** | **2021** | **1990-2021** |
| **Incidence** | | | | | |
| 65-69 years | 3650.5(4631.2,2834.3) | 8117.8(10059.1,6496.3) | 2.95(3.75,2.29) | 2.94(3.65,2.36) | 0.33(0.21,0.45) |
| 70-74 years | 2873.7(3446.2,2279.0) | 7975.3(9323.0,6557.5) | 3.39(4.07,2.69) | 3.87(4.53,3.19) | 0.46(0.42,0.50) |
| 75-79 years | 2305.5(2763.7,1855.4) | 5654.5(6571.1,4657.7) | 3.75(4.49,3.01) | 4.29(4.98,3.53) | 0.38(0.29,0.48) |
| 80-84 years | 1280.2(1572.5,996.1) | 3671.5(4406.4,3010.5) | 3.62(4.45,2.82) | 4.19(5.03,3.44) | 0.45(0.31,0.59) |
| 85-89 years | 465.4(588.2,357.4) | 1667.6(2047.1,1336.0) | 3.08(3.89,2.37) | 3.65(4.48,2.92) | 0.71(0.57,0.85) |
| 90-94 years | 110.3(148.6,78.7) | 566.3(719.0,434.0) | 2.57(3.47,1.84) | 3.17(4.02,2.43) | 0.88(0.79,0.98) |
| 95+ years | 25.2(40.9,14.1) | 164.1(239.9,104.8) | 2.47(4.01,1.39) | 3.01(4.40,1.92) | 0.83(0.73,0.93) |
| **Prevalence** | | | | | |
| 65-69 years | 11237.5(14892.6,8269.5) | 24174.0(31810.6,18400.6) | 9.09(12.05,6.69) | 8.76(11.53,6.67) | 0.36(0.20,0.53) |
| 70-74 years | 8825.2(11715.2,6450.4) | 25047.5(32498.7,19080.3) | 10.42(13.84,7.62) | 12.17(15.79,9.27) | 0.64(0.59,0.69) |
| 75-79 years | 7292.4(9569.5,5421.3) | 17921.6(22727.9,14150.0) | 11.85(15.55,8.81) | 13.59(17.23,10.73) | 0.66(0.53,0.80) |
| 80-84 years | 4151.8(5473.4,2971.2) | 11673.6(14964.7,8830.1) | 11.74(15.47,8.40) | 13.33(17.09,10.08) | 0.60(0.45,0.75) |
| 85-89 years | 1711.5(2328.2,1183.0) | 5874.6(7782.5,4146.3) | 11.33(15.41,7.83) | 12.85(17.02,9.07) | 0.69(0.57,0.80) |
| 90-94 years | 411.4(589.0,270.0) | 2078.9(2879.4,1448.6) | 9.60(13.75,6.30) | 11.62(16.10,8.10) | 0.97(0.86,1.09) |
| 95+ years | 100.1(154.7,57.1) | 625.4(923.8,379.5) | 9.84(15.19,5.61) | 11.47(16.95,6.96) | 0.80(0.65,0.95) |
| **Mortality** | | | | | |
| 65-69 years | 2488.2(2589.9,2386.1) | 6226.0(6720.6,5774.5) | 2.01(2.10,1.93) | 2.26(2.44,2.09) | 0.72(0.60,0.83) |
| 70-74 years | 2110.6(2186.7,2013.4) | 6785.4(7388.9,6213.9) | 2.49(2.58,2.38) | 3.30(3.59,3.02) | 0.79(0.71,0.87) |
| 75-79 years | 1979.6(2053.5,1859.6) | 5519.9(6069.9,4932.6) | 3.22(3.34,3.02) | 4.19(4.60,3.74) | 0.88(0.67,1.09) |
| 80-84 years | 1097.9(1161.5,982.2) | 3692.0(4133.1,3065.1) | 3.10(3.28,2.78) | 4.22(4.72,3.50) | 1.13(0.80,1.47) |
| 85-89 years | 534.1(584.4,457.6) | 1854.2(2105.8,1489.2) | 3.53(3.87,3.03) | 4.06(4.61,3.26) | 1.10(0.77,1.43) |
| 90-94 years | 117.0(128.7,95.7) | 650.7(745.2,501.0) | 2.73(3.00,2.23) | 3.64(4.17,2.80) | 1.46(1.15,1.77) |
| 95+ years | 22.3(24.9,16.9) | 193.4(225.8,140.1) | 2.19(2.45,1.66) | 3.55(4.14,2.57) | 1.82(1.58,2.06) |
| **DALYs** | | | | | |
| 65-69 years | 62758.9(65469.3,60180.8) | 156374.1(169081.2,145377.1) | 50.77(52.96,48.69) | 56.69(61.30,52.70) | 0.71(0.59,0.83) |
| 70-74 years | 44002.8(45700.8,42076.4) | 140469.4(152885.4,129189.1) | 51.98(53.98,49.70) | 68.24(74.27,62.76) | 0.78(0.70,0.85) |
| 75-79 years | 33033.9(34355.7,31227.7) | 91537.1(100417.2,81842.7) | 53.67(55.81,50.73) | 69.41(76.14,62.06) | 0.87(0.66,1.07) |
| 80-84 years | 14575.51(15424.50,13037.71) | 48322.99(53679.18,40379.40) | 41.20(43.60,36.85) | 55.17(61.29,46.10) | 1.09(0.76,1.41) |
| 85-89 years | 5654.09(6167.57,4868.34) | 19511.92(22002.34,15931.57) | 37.42(40.81,32.22) | 42.68(48.12,34.84) | 1.06(0.74,1.37) |
| 90-94 years | 1095.81(1202.52,916.42) | 6042.77(6907.24,4767.30) | 25.57(28.06,21.39) | 33.78(38.61,26.65) | 1.42(1.13,1.72) |
| 95+ years | 202.42(228.60,161.13) | 1688.76(1959.11,1270.62) | 19.88(22.45,15.83) | 30.98(35.94,23.31) | 1.69(1.47,1.90) |

MND, motor neuron disease; DALYs, disability-adjusted life years; EAPCs, estimated annual percentage changes.

Table S5 Age-standardized rates of MND in patients aged 65 years and older across 204 countries in 2021

| **Location** | **Age-standardized incidence rate (per 100,000)** | **Age-standardized prevalence rate (per 100,000)** | **Age-standardized mortality rate (per 100,000)** | **Age-standardized DALYs rate (per 100,000)** |
| --- | --- | --- | --- | --- |
| Afghanistan | 1.08(0.62,1.67) | 1.93(1.04,3.20) | 0.00(0.01,0.00) | 0.46(0.25,0.76) |
| Albania | 1.08(0.67,1.56) | 3.44(2.05,5.24) | 0.34(0.59,0.17) | 7.04(3.90,11.68) |
| Algeria | 0.82(0.47,1.27) | 2.46(1.40,3.95) | 0.01(0.02,0.00) | 0.67(0.40,1.13) |
| American Samoa | 0.99(0.60,1.50) | 2.16(1.22,3.47) | 0.02(0.06,0.01) | 0.70(0.39,1.36) |
| Andorra | 12.85(9.33,16.57) | 51.79(35.71,71.21) | 10.04(18.05,4.87) | 188.10(97.27,331.80) |
| Angola | 1.27(0.72,2.01) | 2.06(1.09,3.42) | 0.00(0.01,0.00) | 0.47(0.25,0.83) |
| Antigua and Barbuda | 2.58(2.02,3.18) | 4.97(3.25,7.09) | 2.66(3.19,2.18) | 52.47(43.08,62.86) |
| Argentina | 3.81(2.95,4.72) | 10.78(7.31,14.96) | 3.72(4.39,3.11) | 72.78(61.34,85.25) |
| Armenia | 0.86(0.49,1.33) | 2.59(1.47,4.11) | 0.40(0.49,0.31) | 8.08(6.49,9.96) |
| Australia | 16.12(13.32,18.96) | 39.66(27.78,54.22) | 13.13(15.36,10.91) | 236.98(199.95,275.74) |
| Austria | 7.39(5.62,9.18) | 32.59(23.76,42.92) | 7.90(9.10,6.69) | 151.09(129.55,173.52) |
| Azerbaijan | 0.84(0.49,1.31) | 2.47(1.40,3.94) | 0.03(0.05,0.01) | 1.06(0.65,1.61) |
| Bahamas | 2.26(1.69,2.88) | 5.21(3.38,7.63) | 2.75(3.65,2.01) | 54.32(39.98,72.04) |
| Bahrain | 0.82(0.47,1.27) | 2.33(1.32,3.72) | 0.03(0.05,0.01) | 1.03(0.64,1.52) |
| Bangladesh | 0.74(0.45,1.11) | 1.64(0.94,2.59) | 0.07(0.18,0.02) | 1.75(0.66,3.73) |
| Barbados | 5.58(4.62,6.52) | 9.11(6.20,12.76) | 6.59(9.62,4.43) | 124.14(83.71,180.82) |
| Belarus | 2.83(2.15,3.55) | 7.37(4.89,10.53) | 3.19(4.13,2.44) | 66.07(50.93,84.66) |
| Belgium | 11.57(8.93,14.28) | 45.52(32.50,61.36) | 12.28(14.96,9.89) | 233.87(191.39,282.69) |
| Belize | 1.52(1.06,2.04) | 3.19(1.95,4.81) | 1.22(1.50,0.96) | 24.15(19.12,29.85) |
| Benin | 1.00(0.57,1.57) | 1.56(0.84,2.61) | 0.00(0.00,0.00) | 0.34(0.18,0.59) |
| Bermuda | 5.31(4.23,6.42) | 12.76(8.60,17.73) | 2.38(3.31,1.74) | 47.75(35.27,65.33) |
| Bhutan | 0.74(0.45,1.11) | 1.84(1.05,2.91) | 0.07(0.15,0.02) | 1.62(0.67,3.17) |
| Bolivia (Plurinational State of) | 2.53(1.96,3.19) | 4.06(2.63,5.96) | 0.75(1.34,0.36) | 14.99(7.71,26.21) |
| Bosnia and Herzegovina | 1.31(0.74,2.06) | 3.76(2.15,6.05) | 1.49(2.13,0.88) | 30.08(18.09,42.64) |
| Botswana | 1.06(0.61,1.66) | 2.23(1.22,3.61) | 0.01(0.04,0.01) | 0.72(0.41,1.29) |
| Brazil | 3.56(2.90,4.23) | 6.23(4.12,8.80) | 4.14(4.53,3.69) | 79.55(71.72,86.83) |
| Brunei Darussalam | 1.64(1.09,2.29) | 5.09(3.17,7.54) | 2.33(4.23,1.06) | 43.92(20.77,79.44) |
| Bulgaria | 1.14(0.67,1.75) | 3.38(1.92,5.34) | 0.72(0.90,0.57) | 15.01(11.96,18.67) |
| Burkina Faso | 1.07(0.62,1.68) | 1.57(0.85,2.62) | 0.00(0.00,0.00) | 0.34(0.17,0.59) |
| Burundi | 1.38(0.79,2.18) | 1.56(0.83,2.63) | 0.00(0.00,0.00) | 0.34(0.18,0.60) |
| Cabo Verde | 0.97(0.55,1.53) | 2.09(1.15,3.43) | 0.00(0.00,0.00) | 0.47(0.25,0.79) |
| Cambodia | 0.98(0.57,1.50) | 1.74(0.95,2.86) | 0.03(0.06,0.01) | 0.93(0.51,1.64) |
| Cameroon | 0.92(0.54,1.43) | 1.45(0.79,2.39) | 0.00(0.00,0.00) | 0.33(0.18,0.56) |
| Canada | 13.95(10.99,16.91) | 48.33(34.59,64.91) | 11.19(12.85,9.55) | 208.94(180.58,238.54) |
| Central African Republic | 1.41(0.80,2.23) | 1.50(0.80,2.51) | 0.00(0.00,0.00) | 0.34(0.18,0.59) |
| Chad | 1.15(0.66,1.81) | 1.69(0.91,2.81) | 0.00(0.00,0.00) | 0.37(0.19,0.64) |
| Chile | 4.78(3.68,5.90) | 14.24(9.87,19.86) | 5.11(6.07,4.29) | 99.39(83.88,117.96) |
| China | 1.00(0.63,1.47) | 2.75(1.59,4.29) | 0.66(0.87,0.42) | 13.32(8.65,17.42) |
| Colombia | 2.71(2.13,3.34) | 5.17(3.42,7.42) | 3.02(3.70,2.39) | 58.87(46.69,72.18) |
| Comoros | 1.35(0.77,2.14) | 2.02(1.08,3.40) | 0.00(0.00,0.00) | 0.45(0.24,0.78) |
| Congo | 1.10(0.62,1.72) | 1.59(0.85,2.63) | 0.00(0.01,0.00) | 0.41(0.22,0.69) |
| Cook Islands | 1.02(0.63,1.51) | 2.72(1.59,4.24) | 0.04(0.09,0.01) | 1.09(0.57,1.84) |
| Costa Rica | 5.78(4.78,6.74) | 10.10(6.88,14.06) | 5.14(6.25,4.14) | 100.08(81.07,121.60) |
| Côted'Ivoire | 0.95(0.55,1.49) | 1.54(0.83,2.53) | 0.00(0.00,0.00) | 0.34(0.18,0.58) |
| Croatia | 2.66(2.01,3.37) | 7.24(4.75,10.28) | 3.96(5.13,3.19) | 78.50(63.27,101.18) |
| Cuba | 3.11(2.47,3.79) | 6.03(4.03,8.61) | 3.74(4.63,2.95) | 72.69(57.59,89.89) |
| Cyprus | 5.27(3.99,6.69) | 21.57(15.27,29.12) | 6.35(10.95,3.50) | 116.13(65.58,198.07) |
| Czechia | 2.42(1.78,3.09) | 7.32(4.75,10.49) | 3.20(3.81,2.69) | 63.89(53.97,75.67) |
| Democratic People's Republic of Korea | 1.70(1.05,2.53) | 3.54(2.02,5.64) | 0.20(0.44,0.09) | 4.75(2.40,9.61) |
| Democratic Republic of the Congo | 1.25(0.70,1.97) | 1.50(0.80,2.50) | 0.00(0.01,0.00) | 0.35(0.18,0.61) |
| Denmark | 11.19(8.42,13.81) | 45.28(32.96,60.73) | 11.94(14.03,10.02) | 224.04(191.33,261.15) |
| Djibouti | 1.33(0.75,2.11) | 2.08(1.12,3.46) | 0.00(0.01,0.00) | 0.47(0.24,0.81) |
| Dominica | 3.94(3.14,4.76) | 6.32(4.17,9.08) | 3.37(5.66,1.77) | 63.40(33.81,104.77) |
| Dominican Republic | 1.74(1.26,2.31) | 4.00(2.51,5.91) | 0.01(0.07,0.01) | 1.13(0.65,2.23) |
| Ecuador | 2.17(1.67,2.74) | 3.99(2.61,5.84) | 2.62(3.34,2.00) | 50.12(38.07,64.03) |
| Egypt | 0.89(0.54,1.34) | 2.07(1.17,3.35) | 0.00(0.00,0.00) | 0.45(0.24,0.76) |
| El Salvador | 1.11(0.70,1.62) | 2.52(1.49,3.95) | 0.49(0.71,0.31) | 10.01(6.49,14.29) |
| Equatorial Guinea | 0.96(0.55,1.50) | 1.80(0.98,2.93) | 0.00(0.01,0.00) | 0.47(0.27,0.79) |
| Eritrea | 1.46(0.84,2.33) | 1.94(1.03,3.25) | 0.00(0.01,0.00) | 0.44(0.23,0.76) |
| Estonia | 1.14(0.72,1.68) | 4.42(2.67,6.69) | 4.44(5.38,3.65) | 89.41(73.90,107.62) |
| Eswatini | 1.19(0.68,1.86) | 2.24(1.22,3.66) | 0.02(0.06,0.01) | 0.84(0.47,1.73) |
| Ethiopia | 1.16(0.64,1.86) | 1.50(0.77,2.55) | 0.00(0.00,0.00) | 0.34(0.17,0.59) |
| Fiji | 1.06(0.63,1.61) | 2.09(1.17,3.39) | 0.02(0.05,0.01) | 0.82(0.50,1.28) |
| Finland | 8.91(6.68,11.19) | 42.47(30.66,55.76) | 14.81(19.13,11.35) | 279.79(217.83,357.20) |
| France | 11.83(9.92,13.63) | 48.84(38.13,61.16) | 11.24(13.82,8.99) | 212.73(173.18,258.51) |
| Gabon | 0.99(0.56,1.55) | 1.73(0.93,2.87) | 0.00(0.01,0.00) | 0.45(0.25,0.78) |
| Gambia | 1.06(0.61,1.65) | 1.63(0.88,2.69) | 0.00(0.00,0.00) | 0.36(0.19,0.61) |
| Georgia | 0.92(0.56,1.37) | 2.73(1.59,4.25) | 0.60(0.73,0.49) | 12.21(10.00,14.76) |
| Germany | 8.84(6.73,10.83) | 37.04(27.10,49.50) | 9.10(10.51,7.73) | 174.67(151.50,199.62) |
| Ghana | 0.90(0.53,1.38) | 1.55(0.85,2.55) | 0.00(0.00,0.00) | 0.36(0.20,0.60) |
| Greece | 5.07(3.73,6.37) | 21.44(15.12,28.77) | 6.78(8.06,5.61) | 129.70(108.04,153.34) |
| Greenland | 6.51(4.39,9.04) | 26.60(17.69,37.85) | 2.03(3.22,0.51) | 44.48(15.93,67.03) |
| Grenada | 2.16(1.66,2.75) | 3.58(2.27,5.27) | 2.27(2.73,1.82) | 45.14(36.21,54.24) |
| Guam | 0.92(0.54,1.39) | 2.53(1.44,4.00) | 0.02(0.05,0.01) | 0.75(0.43,1.33) |
| Guatemala | 1.21(0.77,1.73) | 2.44(1.44,3.83) | 0.54(0.65,0.44) | 10.90(8.96,13.14) |
| Guinea | 1.04(0.60,1.62) | 1.54(0.83,2.57) | 0.00(0.00,0.00) | 0.34(0.18,0.58) |
| Guinea-Bissau | 1.08(0.62,1.67) | 1.47(0.78,2.47) | 0.00(0.00,0.00) | 0.33(0.17,0.56) |
| Guyana | 2.00(1.52,2.56) | 3.01(1.92,4.52) | 2.18(2.86,1.59) | 42.99(31.58,56.24) |
| Haiti | 1.77(1.29,2.33) | 2.48(1.52,3.80) | 0.38(0.98,0.11) | 8.01(2.71,19.83) |
| Honduras | 1.83(1.36,2.37) | 3.22(2.02,4.79) | 1.32(2.60,0.53) | 26.57(11.11,50.81) |
| Hungary | 2.46(1.83,3.12) | 6.71(4.37,9.64) | 3.85(4.63,3.22) | 78.51(66.03,93.81) |
| Iceland | 9.77(6.98,12.46) | 48.67(35.02,65.52) | 8.66(10.28,7.16) | 163.54(138.93,192.39) |
| India | 0.76(0.47,1.12) | 1.54(0.87,2.46) | 0.12(0.18,0.07) | 2.69(1.61,3.90) |
| Indonesia | 0.68(0.40,1.04) | 1.32(0.72,2.18) | 0.07(0.12,0.04) | 1.54(0.95,2.43) |
| Iran (Islamic Republic of) | 1.04(0.67,1.49) | 3.06(1.85,4.66) | 0.28(0.51,0.15) | 5.97(3.46,10.23) |
| Iraq | 0.94(0.54,1.47) | 2.55(1.42,4.15) | 0.00(0.00,0.00) | 0.55(0.29,0.93) |
| Ireland | 16.94(12.82,20.84) | 63.00(44.67,86.65) | 14.85(18.46,11.56) | 279.65(220.75,344.43) |
| Israel | 5.73(4.34,7.15) | 23.38(16.38,31.07) | 6.18(7.27,5.08) | 115.31(97.07,134.32) |
| Italy | 9.84(7.64,12.04) | 37.15(27.04,49.51) | 9.35(10.77,7.91) | 176.66(152.54,202.77) |
| Jamaica | 1.74(1.23,2.36) | 3.69(2.31,5.53) | 2.68(3.60,1.89) | 52.20(36.77,70.34) |
| Japan | 5.67(4.45,6.91) | 19.59(14.08,26.14) | 6.68(7.44,5.70) | 122.33(106.80,134.88) |
| Jordan | 0.91(0.52,1.43) | 2.44(1.37,3.97) | 0.18(0.29,0.11) | 4.08(2.62,6.20) |
| Kazakhstan | 0.98(0.55,1.56) | 3.16(1.79,5.04) | 0.00(0.00,0.00) | 0.72(0.40,1.18) |
| Kenya | 0.91(0.51,1.46) | 1.34(0.69,2.25) | 0.00(0.00,0.00) | 0.32(0.17,0.55) |
| Kiribati | 3.25(2.58,3.96) | 4.05(2.61,5.93) | 0.89(1.60,0.44) | 19.90(10.25,34.88) |
| Kuwait | 1.15(0.77,1.61) | 3.80(2.41,5.61) | 1.04(1.37,0.71) | 20.50(14.26,27.05) |
| Kyrgyzstan | 0.91(0.52,1.42) | 2.39(1.34,3.82) | 0.32(0.41,0.25) | 6.85(5.38,8.57) |
| Lao People's Democratic Republic | 1.00(0.60,1.53) | 1.94(1.08,3.16) | 0.03(0.07,0.01) | 0.96(0.49,1.85) |
| Latvia | 1.49(1.00,2.07) | 5.03(3.14,7.43) | 3.94(4.80,3.17) | 81.35(65.78,98.29) |
| Lebanon | 0.87(0.52,1.34) | 2.63(1.51,4.17) | 0.52(0.89,0.28) | 10.47(5.91,17.35) |
| Lesotho | 1.30(0.75,2.04) | 2.17(1.19,3.58) | 0.01(0.02,0.00) | 0.63(0.36,1.09) |
| Liberia | 1.02(0.59,1.60) | 1.40(0.76,2.34) | 0.00(0.00,0.00) | 0.31(0.16,0.54) |
| Libya | 0.78(0.45,1.18) | 2.22(1.26,3.53) | 0.01(0.03,0.01) | 0.75(0.45,1.26) |
| Lithuania | 4.00(3.12,4.88) | 11.05(7.44,15.41) | 7.08(8.70,5.73) | 145.53(118.94,176.79) |
| Luxembourg | 7.69(5.62,9.69) | 35.53(25.75,47.39) | 7.83(9.36,6.49) | 148.70(124.66,175.67) |
| Madagascar | 1.58(0.90,2.47) | 2.23(1.20,3.73) | 0.00(0.00,0.00) | 0.49(0.25,0.85) |
| Malawi | 1.46(0.83,2.32) | 1.90(1.02,3.18) | 0.00(0.00,0.00) | 0.42(0.22,0.74) |
| Malaysia | 0.80(0.46,1.24) | 1.88(1.06,3.06) | 0.10(0.20,0.04) | 2.26(1.13,4.19) |
| Maldives | 0.93(0.60,1.32) | 2.02(1.20,3.13) | 0.99(1.75,0.50) | 18.92(9.65,33.24) |
| Mali | 2.97(2.26,3.80) | 3.86(2.45,5.67) | 0.00(0.00,0.00) | 0.83(0.48,1.31) |
| Malta | 7.77(5.85,9.62) | 29.81(21.03,40.20) | 7.68(9.35,6.25) | 145.08(119.18,175.21) |
| Marshall Islands | 1.07(0.68,1.58) | 1.69(0.96,2.72) | 0.02(0.06,0.00) | 0.66(0.32,1.15) |
| Mauritania | 1.10(0.64,1.68) | 1.98(1.08,3.22) | 0.00(0.00,0.00) | 0.44(0.23,0.74) |
| Mauritius | 1.10(0.72,1.55) | 2.73(1.63,4.18) | 2.96(3.50,2.49) | 58.10(49.14,68.64) |
| Mexico | 2.02(1.52,2.59) | 4.25(2.75,6.17) | 2.09(2.39,1.82) | 41.05(35.71,47.03) |
| Micronesia (Federated States of) | 1.10(0.68,1.61) | 1.66(0.94,2.66) | 0.02(0.04,0.00) | 0.60(0.31,1.06) |
| Monaco | 3.11(2.14,4.16) | 19.72(13.96,26.55) | 2.59(4.51,1.39) | 50.33(28.77,84.66) |
| Mongolia | 1.10(0.70,1.58) | 2.92(1.70,4.49) | 0.23(0.37,0.13) | 5.00(3.09,7.72) |
| Montenegro | 1.17(0.67,1.79) | 3.58(2.05,5.65) | 0.08(0.12,0.05) | 2.31(1.62,3.14) |
| Morocco | 0.92(0.53,1.42) | 2.27(1.26,3.66) | 0.01(0.02,0.00) | 0.60(0.35,1.04) |
| Mozambique | 1.54(0.88,2.44) | 2.06(1.10,3.42) | 0.00(0.01,0.00) | 0.46(0.24,0.80) |
| Myanmar | 1.02(0.61,1.55) | 2.11(1.18,3.45) | 0.04(0.09,0.02) | 1.22(0.69,2.22) |
| Namibia | 1.08(0.62,1.69) | 2.20(1.21,3.59) | 0.02(0.05,0.01) | 0.76(0.43,1.48) |
| Nauru | 1.00(0.63,1.45) | 1.68(0.95,2.69) | 0.03(0.06,0.01) | 0.74(0.39,1.25) |
| Nepal | 0.80(0.48,1.19) | 1.64(0.92,2.63) | 0.05(0.13,0.01) | 1.35(0.54,2.83) |
| Netherlands | 13.85(10.35,17.11) | 53.99(38.36,73.91) | 13.34(15.44,11.25) | 245.80(210.15,281.75) |
| New Zealand | 15.63(12.68,18.71) | 39.56(27.26,55.29) | 11.78(13.84,9.79) | 218.09(183.19,254.62) |
| Nicaragua | 1.25(0.81,1.78) | 2.72(1.62,4.27) | 0.48(0.82,0.25) | 10.08(5.44,16.76) |
| Niger | 1.24(0.71,1.95) | 1.72(0.92,2.87) | 0.00(0.00,0.00) | 0.37(0.19,0.64) |
| Nigeria | 0.75(0.43,1.19) | 1.30(0.69,2.15) | 0.00(0.00,0.00) | 0.30(0.16,0.51) |
| Niue | 1.19(0.76,1.72) | 2.54(1.48,4.01) | 0.04(0.08,0.01) | 1.06(0.57,1.69) |
| North Macedonia | 1.18(0.68,1.81) | 3.27(1.86,5.20) | 0.99(1.45,0.58) | 19.76(11.97,28.56) |
| Northern Mariana Islands | 0.96(0.55,1.48) | 2.48(1.38,4.01) | 0.02(0.06,0.01) | 0.76(0.41,1.39) |
| Norway | 12.49(9.50,15.62) | 52.54(38.49,70.44) | 12.24(13.54,10.85) | 233.94(210.78,256.86) |
| Oman | 1.40(0.99,1.91) | 3.20(2.00,4.81) | 0.23(0.42,0.11) | 5.29(2.90,9.03) |
| Pakistan | 0.75(0.46,1.11) | 1.57(0.87,2.55) | 0.11(0.21,0.04) | 2.51(1.14,4.38) |
| Palau | 1.12(0.73,1.59) | 2.21(1.29,3.46) | 0.01(0.02,0.00) | 0.66(0.39,1.08) |
| Palestine | 1.02(0.58,1.60) | 2.22(1.22,3.63) | 0.29(0.47,0.16) | 5.99(3.56,9.59) |
| Panama | 1.66(1.21,2.20) | 4.10(2.61,5.98) | 2.20(2.77,1.64) | 42.78(31.94,53.83) |
| Papua New Guinea | 0.94(0.56,1.43) | 1.61(0.89,2.63) | 0.00(0.01,0.00) | 0.38(0.20,0.68) |
| Paraguay | 1.74(1.06,2.60) | 4.00(2.30,6.41) | 0.30(0.43,0.19) | 6.53(4.36,8.95) |
| Peru | 1.35(0.93,1.85) | 3.09(1.92,4.66) | 1.03(1.74,0.52) | 20.10(10.52,33.54) |
| Philippines | 0.72(0.43,1.10) | 1.52(0.84,2.47) | 0.10(0.12,0.07) | 2.16(1.61,2.69) |
| Poland | 3.58(2.79,4.37) | 9.34(6.22,12.97) | 5.11(5.80,4.52) | 102.72(91.08,115.89) |
| Portugal | 6.94(5.34,8.59) | 27.55(19.85,36.47) | 7.42(8.70,6.19) | 142.41(120.43,166.43) |
| Puerto Rico | 3.55(2.81,4.29) | 8.11(5.43,11.25) | 3.02(3.80,2.39) | 58.58(46.93,73.40) |
| Qatar | 0.77(0.43,1.20) | 2.62(1.50,4.20) | 0.01(0.01,0.00) | 0.66(0.39,1.06) |
| Republic of Korea | 1.76(1.08,2.58) | 7.61(4.75,11.21) | 1.89(2.63,1.15) | 35.61(22.08,49.21) |
| Republic of Moldova | 1.04(0.62,1.56) | 2.83(1.63,4.46) | 1.86(2.25,1.52) | 39.21(32.26,47.19) |
| Romania | 1.13(0.70,1.63) | 3.68(2.21,5.58) | 1.27(1.56,1.03) | 26.03(21.08,31.89) |
| Russian Federation | 1.81(1.33,2.36) | 5.03(3.20,7.27) | 3.90(4.36,3.50) | 81.12(73.04,90.29) |
| Rwanda | 1.19(0.67,1.88) | 1.57(0.84,2.62) | 0.00(0.00,0.00) | 0.35(0.18,0.61) |
| Saint Kitts and Nevis | 3.44(2.79,4.11) | 5.80(3.78,8.29) | 4.04(5.16,3.03) | 80.63(60.22,103.25) |
| Saint Lucia | 3.18(2.54,3.86) | 5.75(3.84,8.26) | 3.99(5.04,3.02) | 75.38(56.94,95.50) |
| Saint Vincent and the Grenadines | 2.20(1.69,2.77) | 4.00(2.59,5.78) | 2.17(2.63,1.77) | 41.79(34.19,50.68) |
| Samoa | 1.07(0.67,1.57) | 2.12(1.22,3.40) | 0.02(0.06,0.00) | 0.71(0.36,1.29) |
| San Marino | 2.30(1.42,3.39) | 14.44(9.46,20.76) | 1.09(2.01,0.49) | 22.68(11.80,39.07) |
| Sao Tome and Principe | 1.60(1.19,2.06) | 2.35(1.48,3.53) | 0.00(0.01,0.00) | 0.54(0.32,0.83) |
| Saudi Arabia | 0.82(0.50,1.21) | 2.42(1.42,3.75) | 0.20(0.39,0.09) | 4.55(2.30,8.39) |
| Senegal | 1.03(0.60,1.62) | 1.73(0.93,2.87) | 0.00(0.00,0.00) | 0.39(0.21,0.66) |
| Serbia | 1.24(0.72,1.91) | 3.61(2.06,5.76) | 0.25(0.35,0.16) | 5.66(3.81,7.76) |
| Seychelles | 1.23(0.85,1.66) | 2.73(1.68,4.13) | 0.04(0.07,0.02) | 1.34(0.84,2.00) |
| Sierra Leone | 1.04(0.60,1.65) | 1.45(0.78,2.44) | 0.00(0.00,0.00) | 0.32(0.17,0.55) |
| Singapore | 2.02(1.47,2.63) | 7.23(4.81,10.15) | 2.98(3.57,2.46) | 55.78(46.48,66.59) |
| Slovakia | 1.19(0.74,1.73) | 4.10(2.44,6.24) | 0.74(1.03,0.49) | 15.60(10.53,21.36) |
| Slovenia | 1.24(0.71,1.93) | 4.49(2.61,7.06) | 1.31(1.60,1.07) | 26.24(21.66,31.99) |
| Solomon Islands | 1.04(0.62,1.57) | 1.54(0.85,2.53) | 0.00(0.01,0.00) | 0.37(0.19,0.67) |
| Somalia | 1.56(0.88,2.49) | 1.40(0.74,2.35) | 0.00(0.00,0.00) | 0.30(0.15,0.53) |
| South Africa | 0.98(0.55,1.56) | 2.18(1.16,3.58) | 0.01(0.04,0.01) | 0.71(0.41,1.29) |
| South Sudan | 1.37(0.78,2.17) | 1.83(0.97,3.06) | 0.00(0.00,0.00) | 0.40(0.21,0.70) |
| Spain | 8.69(6.52,10.65) | 34.95(25.41,46.74) | 8.69(10.61,6.86) | 165.33(132.68,201.02) |
| Sri Lanka | 0.92(0.58,1.34) | 2.09(1.22,3.26) | 0.41(0.63,0.22) | 8.16(4.47,12.53) |
| Sudan | 0.81(0.46,1.25) | 1.62(0.90,2.65) | 0.00(0.01,0.00) | 0.41(0.23,0.68) |
| Suriname | 2.04(1.54,2.60) | 3.76(2.40,5.58) | 1.40(2.42,0.70) | 27.75(14.23,47.17) |
| Sweden | 14.09(10.90,17.24) | 56.30(40.74,75.22) | 11.95(13.90,10.05) | 224.81(191.41,260.10) |
| Switzerland | 10.64(7.92,13.30) | 45.44(32.35,60.75) | 9.66(11.40,7.97) | 179.30(151.30,208.64) |
| Syrian Arab Republic | 0.97(0.56,1.50) | 2.39(1.33,3.89) | 0.01(0.02,0.00) | 0.63(0.36,1.06) |
| Taiwan (Province of China) | 2.06(1.51,2.68) | 5.96(3.90,8.55) | 1.56(2.03,1.19) | 31.69(24.22,40.97) |
| Tajikistan | 0.95(0.54,1.49) | 2.17(1.20,3.57) | 0.01(0.03,0.00) | 0.70(0.38,1.18) |
| Thailand | 0.93(0.54,1.46) | 2.47(1.40,3.98) | 0.09(0.16,0.04) | 2.34(1.33,3.74) |
| Timor-Leste | 0.88(0.52,1.35) | 1.69(0.95,2.76) | 0.02(0.05,0.01) | 0.70(0.38,1.32) |
| Togo | 0.99(0.57,1.55) | 1.45(0.78,2.42) | 0.00(0.00,0.00) | 0.32(0.17,0.55) |
| Tokelau | 0.99(0.60,1.46) | 1.89(1.07,3.03) | 0.03(0.07,0.01) | 0.83(0.42,1.39) |
| Tonga | 1.09(0.66,1.64) | 2.32(1.32,3.70) | 0.02(0.05,0.00) | 0.79(0.41,1.35) |
| Trinidad and Tobago | 2.12(1.61,2.71) | 4.21(2.74,6.12) | 2.72(3.71,1.92) | 53.12(37.66,72.46) |
| Tunisia | 0.89(0.51,1.37) | 2.62(1.50,4.19) | 0.01(0.02,0.00) | 0.72(0.43,1.18) |
| Turkey | 2.85(2.16,3.54) | 7.28(4.83,10.31) | 2.96(4.95,1.67) | 58.81(33.78,96.71) |
| Turkmenistan | 0.89(0.55,1.31) | 2.67(1.56,4.09) | 0.00(0.00,0.00) | 0.57(0.32,0.94) |
| Tuvalu | 1.01(0.61,1.51) | 1.65(0.93,2.66) | 0.02(0.05,0.00) | 0.60(0.30,1.04) |
| Uganda | 1.15(0.65,1.83) | 1.59(0.86,2.64) | 0.00(0.01,0.00) | 0.37(0.19,0.64) |
| Ukraine | 0.88(0.53,1.34) | 2.55(1.43,4.08) | 0.40(0.53,0.29) | 9.32(6.80,12.38) |
| United Arab Emirates | 0.87(0.53,1.27) | 2.77(1.63,4.34) | 1.86(3.22,1.00) | 35.21(19.23,60.53) |
| United Kingdom | 16.54(12.90,20.09) | 54.99(39.75,74.91) | 14.09(15.33,12.63) | 253.39(231.11,273.73) |
| United Republic of Tanzania | 1.24(0.70,1.98) | 1.84(0.98,3.07) | 0.00(0.00,0.00) | 0.42(0.21,0.72) |
| United States of America | 12.22(10.45,13.93) | 34.44(28.18,41.72) | 10.00(10.70,8.93) | 187.14(170.11,198.66) |
| United States Virgin Islands | 5.87(4.61,7.23) | 10.77(7.13,15.40) | 2.80(4.86,1.50) | 52.19(29.11,88.24) |
| Uruguay | 6.74(5.44,7.97) | 16.81(11.74,23.03) | 7.45(8.85,6.20) | 145.09(121.54,171.48) |
| Uzbekistan | 0.96(0.54,1.49) | 2.37(1.30,3.84) | 0.00(0.00,0.00) | 0.52(0.28,0.88) |
| Vanuatu | 1.12(0.67,1.67) | 1.83(1.02,2.96) | 0.01(0.02,0.00) | 0.46(0.24,0.83) |
| Venezuela (Bolivarian Republic of) | 2.07(1.57,2.62) | 3.93(2.56,5.77) | 1.82(2.56,1.17) | 35.69(23.22,49.89) |
| Viet Nam | 0.97(0.60,1.44) | 2.12(1.22,3.37) | 0.07(0.15,0.03) | 1.79(1.00,3.22) |
| Yemen | 0.84(0.48,1.30) | 1.53(0.83,2.51) | 0.00(0.01,0.00) | 0.37(0.21,0.64) |
| Zambia | 1.34(0.76,2.11) | 2.01(1.09,3.34) | 0.00(0.00,0.00) | 0.46(0.25,0.78) |
| Zimbabwe | 1.16(0.75,1.68) | 1.84(1.06,2.94) | 0.18(0.36,0.08) | 4.23(1.93,7.85) |

MND, motor neuron disease; DALYs, disability-adjusted life years.

Table S6 Trends in age-standardized rates of EAPCs among older people with MND in 204 countries, 1990-2021

| **Countries and territories** | **Age-standardized incidence rate (per 100,000)** | **Age-standardized prevalence rate (per 100,000)** | **Age-standardized mortality rate (per 100,000)** | **Age-standardized DALYs rate (per 100,000)** |
| --- | --- | --- | --- | --- |
| Afghanistan | -0.44(-0.56, -0.31) | -0.15(-0.23, -0.07) | 9.60(8.50,10.72) | 0.16(0.04,0.28) |
| Albania | -0.21(-0.30, -0.13) | 0.45(0.36,0.55) | 2.73(2.46,3.00) | 2.32(2.08,2.55) |
| Algeria | -0.36(-0.44, -0.27) | -0.04(-0.10,0.03) | 6.72(6.26,7.19) | 0.69(0.59,0.78) |
| American Samoa | -1.52(-1.69, -1.36) | -1.31(-1.44, -1.18) | -5.91(-6.89, -4.91) | -4.38(-5.03, -3.72) |
| Andorra | 1.05(0.99,1.11) | 0.96(0.91,1.01) | 1.48(1.24,1.73) | 1.33(1.09,1.57) |
| Angola | -0.60(-0.72, -0.48) | -0.04(-0.10,0.03) | -5.23(-6.21, -4.25) | -0.86(-1.04, -0.69) |
| Antigua and Barbuda | 1.84(1.76,1.91) | 1.46(1.41,1.51) | 10.98(7.45,14.62) | 9.60(6.68,12.60) |
| Argentina | 1.46(1.28,1.64) | 1.64(1.48,1.80) | 8.09(5.91,10.31) | 7.60(5.56,9.67) |
| Armenia | -0.44(-0.52, -0.36) | 0.20(0.06,0.34) | 4.25(3.48,5.02) | 3.67(2.98,4.37) |
| Australia | 1.13(1.03,1.22) | 1.34(1.22,1.46) | 0.77(0.43,1.12) | 0.66(0.34,0.99) |
| Austria | 1.71(1.54,1.88) | 1.69(1.53,1.85) | 2.02(1.82,2.21) | 1.96(1.78,2.14) |
| Azerbaijan | -0.64(-0.75, -0.52) | 0.13(-0.06,0.32) | 7.56(6.82,8.30) | 2.43(2.10,2.77) |
| Bahamas | 1.36(1.26,1.46) | 1.13(1.02,1.23) | 12.59(8.73,16.59) | 10.61(7.62,13.68) |
| Bahrain | -0.24(-0.33, -0.16) | 0.08(0.02,0.14) | 1.14(0.76,1.51) | 0.53(0.36,0.70) |
| Bangladesh | -0.26(-0.33, -0.20) | 0.30(0.22,0.37) | 6.56(6.37,6.74) | 3.96(3.72,4.20) |
| Barbados | 2.63(2.31,2.95) | 2.08(1.79,2.37) | 12.83(9.15,16.64) | 11.73(8.51,15.05) |
| Belarus | 1.54(1.43,1.65) | 1.47(1.38,1.56) | 6.74(6.31,7.17) | 6.61(6.17,7.06) |
| Belgium | -0.23(-0.43, -0.04) | 0.17(0.07,0.28) | 0.75(-0.37,1.88) | 0.85(-0.14,1.84) |
| Belize | 0.07(0.01,0.14) | 0.23(0.19,0.27) | 11.61(7.62,15.75) | 9.28(6.28,12.37) |
| Benin | -0.33(-0.42, -0.24) | -0.08(-0.15, -0.01) | -0.19(-0.40,0.02) | -0.10(-0.17, -0.03) |
| Bermuda | 0.19(0.15,0.24) | 0.28(0.24,0.33) | 8.11(5.51,10.78) | 6.56(4.55,8.61) |
| Bhutan | -0.32(-0.40, -0.25) | 0.42(0.36,0.48) | 7.97(7.72,8.22) | 4.38(4.20,4.57) |
| Bolivia (Plurinational State of) | 1.82(1.71,1.94) | 1.71(1.62,1.80) | 11.73(10.48,13.00) | 9.65(8.79,10.52) |
| Bosnia and Herzegovina | 0.08(-0.01,0.18) | 0.95(0.85,1.04) | 3.40(3.15,3.65) | 3.22(2.98,3.46) |
| Botswana | -0.54(-0.63, -0.45) | -0.01(-0.08,0.06) | -5.10(-5.62, -4.57) | -3.12(-3.50,-2.74) |
| Brazil | 2.40(2.23,2.57) | 2.18(2.05,2.31) | 3.76(3.63,3.89) | 3.64(3.52,3.76) |
| Brunei Darussalam | -0.19(-0.28, -0.10) | 0.14(0.06,0.22) | 1.91(1.73,2.10) | 1.69(1.51,1.87) |
| Bulgaria | -0.25(-0.36, -0.15) | 0.20(0.11,0.30) | 3.88(3.61,4.15) | 3.61(3.35,3.87) |
| Burkina Faso | -0.27(-0.37, -0.17) | -0.01(-0.09,0.08) | -0.54(-0.69, -0.38) | -0.03(-0.10,0.05) |
| Burundi | -0.14(-0.23, -0.04) | -0.26(-0.33, -0.19) | -7.43(-8.57, -6.27) | -1.05(-1.23, -0.87) |
| Cabo Verde | -0.79(-0.89, -0.69) | -0.08(-0.15, -0.01) | 2.02(1.76,2.27) | -0.01(-0.09,0.06) |
| Cambodia | -0.65(-0.77, -0.54) | 0.05(-0.04,0.14) | 2.80(2.66,2.94) | 1.38(1.28,1.48) |
| Cameroon | -0.33(-0.41, -0.24) | -0.19(-0.26, -0.12) | -0.38(-0.62, -0.14) | -0.23(-0.30, -0.16) |
| Canada | 0.96(0.87,1.05) | 1.15(1.06,1.24) | 0.68(0.33,1.03) | 0.64(0.31,0.96) |
| Central African Republic | -0.23(-0.32, -0.13) | -0.29(-0.36, -0.22) | -6.91(-7.77, -6.03) | -1.24(-1.40, -1.08) |
| Chad | -0.28(-0.39, -0.18) | 0.06(-0.01,0.12) | 1.51(1.25,1.77) | 0.07(0.01,0.14) |
| Chile | 1.54(1.43,1.64) | 1.90(1.77,2.02) | 4.21(3.14,5.30) | 3.96(2.97,4.96) |
| China | -0.72(-0.87, -0.58) | 0.38(0.27,0.48) | 0.69(0.25,1.12) | 0.62(0.19,1.05) |
| Colombia | 2.92(2.74,3.11) | 2.57(2.43,2.71) | 3.53(3.26,3.80) | 3.50(3.23,3.77) |
| Comoros | -0.33(-0.42, -0.24) | -0.28(-0.36, -0.21) | -6.07(-6.92, -5.20) | -0.94(-1.08, -0.80) |
| Congo | -0.42(-0.52, -0.32) | -0.15(-0.22, -0.08) | -6.24(-7.21, -5.26) | -2.19(-2.57, -1.82) |
| Cook Islands | -1.29(-1.40, -1.18) | -0.71(-0.80, -0.61) | -0.67(-0.83, -0.52) | -0.96(-1.08, -0.84) |
| Costa Rica | 3.08(2.61,3.54) | 2.72(2.38,3.07) | 3.73(3.24,4.23) | 3.72(3.23,4.23) |
| Côte d'Ivoire | -0.26(-0.34, -0.18) | -0.16(-0.24, -0.07) | -0.90(-1.13, -0.66) | -0.22(-0.30, -0.14) |
| Croatia | 1.48(1.40,1.55) | 1.46(1.36,1.57) | 3.16(2.82,3.49) | 3.11(2.79,3.42) |
| Cuba | 2.04(1.87,2.20) | 1.78(1.69,1.88) | 12.28(8.49,16.19) | 11.01(7.82,14.31) |
| Cyprus | 0.51(0.46,0.57) | 0.70(0.62,0.78) | 0.20(-0.01,0.41) | 0.46(0.20,0.72) |
| Czechia | 1.55(1.44,1.66) | 1.47(1.36,1.57) | 2.95(2.52,3.38) | 2.80(2.37,3.23) |
| Democratic People's Republic of Korea | -0.51(-0.66,-0.37) | -0.44(-0.56,-0.33) | 0.13(-0.12,0.39) | 0.04(-0.16,0.24) |
| Democratic Republic of the Congo | -0.28(-0.40,-0.17) | -0.41(-0.51,-0.32) | -6.18(-7.79,-4.54) | -1.40(-1.70,-1.10) |
| Denmark | 0.51(0.45,0.56) | 0.67(0.59,0.75) | 1.22(0.98,1.46) | 1.01(0.77,1.25) |
| Djibouti | -0.33(-0.43,-0.24) | -0.25(-0.33,-0.16) | -6.02(-6.78,-5.25) | -1.06(-1.23,-0.89) |
| Dominica | 2.63(2.36,2.89) | 2.20(1.98,2.43) | 11.82(9.02,14.69) | 10.82(8.35,13.34) |
| Dominican Republic | 0.82(0.69,0.95) | 1.07(0.97,1.18) | 1.51(-0.14,3.19) | 1.15(0.64,1.66) |
| Ecuador | 2.91(2.66,3.15) | 2.50(2.30,2.69) | 26.06(18.32,34.30) | 16.60(12.61,20.73) |
| Egypt | -0.40(-0.48,-0.32) | 0.19(0.14,0.25) | -16.31(-19.05,-13.47) | -3.56(-4.30,-2.81) |
| El Salvador | -0.47(-0.56,-0.37) | -0.06(-0.13,0.01) | 3.73(3.55,3.91) | 3.37(3.22,3.52) |
| Equatorial Guinea | -1.20(-1.35,-1.05) | 0.45(0.28,0.61) | -2.36(-2.96,-1.76) | -0.34(-0.41,-0.27) |
| Eritrea | -0.41(-0.52,-0.30) | -0.21(-0.30,-0.12) | -4.87(-5.58,-4.16) | -0.84(-0.96,-0.72) |
| Estonia | -0.14(-0.21,-0.07) | 0.41(0.32,0.50) | 5.37(2.39,8.44) | 5.01(2.31,7.79) |
| Eswatini | -0.45(-0.55,-0.34) | -0.10(-0.17,-0.03) | -3.47(-3.88,-3.06) | -2.52(-2.83,-2.21) |
| Ethiopia | -0.75(-0.94,-0.56) | -0.12(-0.27,0.02) | -4.83(-6.03,-3.62) | -0.64(-0.84,-0.45) |
| Fiji | -0.41(-0.51,-0.32) | -0.10(-0.18,-0.02) | 4.58(3.81,5.35) | 1.55(1.20,1.91) |
| Finland | -1.24(-1.47,-1.01) | -0.61(-0.81,-0.40) | 0.96(0.81,1.10) | 0.83(0.70,0.96) |
| France | 0.88(0.84,0.92) | 1.07(1.01,1.13) | 0.88(0.79,0.96) | 0.82(0.75,0.88) |
| Gabon | -0.38(-0.48,-0.28) | -0.28(-0.35,-0.20) | -5.99(-6.83,-5.13) | -2.31(-2.65,-1.98) |
| Gambia | -0.25(-0.36,-0.15) | -0.22(-0.30,-0.14) | 0.05(-0.13,0.22) | -0.23(-0.31,-0.15) |
| Georgia | 0.05(-0.00,0.10) | 0.34(0.13,0.55) | 21.59(18.28,24.99) | 12.86(10.93,14.84) |
| Germany | 1.59(1.49,1.70) | 1.60(1.49,1.72) | 1.57(1.25,1.90) | 1.54(1.27,1.81) |
| Ghana | -0.54(-0.62,-0.45) | -0.07(-0.14,0.01) | 0.46(0.18,0.74) | -0.04(-0.13,0.04) |
| Greece | 2.36(2.17,2.55) | 2.03(1.82,2.24) | 3.12(2.97,3.28) | 3.18(3.02,3.34) |
| Greenland | -0.33(-0.45,-0.21) | 0.46(0.37,0.55) | 1.11(0.93,1.30) | 1.01(0.85,1.17) |
| Grenada | 0.14(-0.11,0.39) | 0.38(0.21,0.55) | 11.27(7.54,15.12) | 9.97(6.78,13.26) |
| Guam | -5.22(-5.89,-4.54) | -4.00(-4.52,-3.48) | -12.59(-14.04,-11.13) | -10.16(-11.20,-9.10) |
| Guatemala | -0.32(-0.41,-0.23) | -0.01(-0.08,0.06) | 2.84(2.58,3.09) | 2.72(2.49,2.96) |
| Guinea | -0.25(-0.34,-0.16) | -0.19(-0.27,-0.11) | 0.72(0.57,0.86) | -0.17(-0.25,-0.08) |
| Guinea-Bissau | -0.29(-0.38,-0.20) | -0.21(-0.29,-0.14) | -0.09(-0.28,0.09) | -0.23(-0.31,-0.16) |
| Guyana | 1.30(1.21,1.39) | 1.38(1.32,1.44) | 11.71(8.30,15.23) | 10.62(7.68,13.64) |
| Haiti | 0.39(0.34,0.44) | 0.29(0.24,0.34) | 7.90(6.96,8.85) | 6.57(5.88,7.26) |
| Honduras | 1.19(1.08,1.29) | 1.13(1.05,1.22) | 5.10(4.92,5.29) | 4.94(4.78,5.10) |
| Hungary | 1.63(1.51,1.76) | 1.60(1.47,1.73) | 3.86(3.49,4.23) | 3.78(3.40,4.15) |
| Iceland | 0.57(0.52,0.62) | 0.73(0.68,0.77) | 1.11(0.84,1.39) | 0.97(0.70,1.23) |
| India | 0.13(0.05,0.21) | 0.87(0.78,0.96) | 5.22(5.01,5.44) | 4.20(3.97,4.43) |
| Indonesia | -0.74(-0.88,-0.60) | -0.12(-0.23,-0.01) | 2.26(2.04,2.49) | 1.55(1.40,1.71) |
| Iran (Islamic Republic of) | -0.07(-0.14,-0.01) | 0.51(0.44,0.57) | 4.67(4.52,4.82) | 4.05(3.91,4.20) |
| Iraq | -0.54(-0.64,-0.45) | 0.01(-0.07,0.09) | 5.16(5.05,5.27) | 0.05(-0.03,0.13) |
| Ireland | 1.62(1.55,1.69) | 1.80(1.74,1.87) | 1.29(1.13,1.44) | 1.22(1.06,1.38) |
| Israel | 0.66(0.63,0.69) | 0.76(0.73,0.80) | 0.82(0.68,0.97) | 0.73(0.59,0.87) |
| Italy | 1.93(1.81,2.05) | 1.73(1.54,1.92) | 2.57(2.19,2.95) | 2.33(1.99,2.67) |
| Jamaica | 0.60(0.51,0.70) | 0.63(0.56,0.70) | 12.73(8.80,16.80) | 11.07(7.77,14.46) |
| Japan | 1.49(1.42,1.57) | 1.75(1.54,1.97) | 1.96(1.84,2.08) | 1.69(1.57,1.81) |
| Jordan | -0.40(-0.50,-0.29) | 0.02(-0.05,0.08) | 4.61(4.10,5.13) | 3.65(3.22,4.07) |
| Kazakhstan | -0.55(-0.63,-0.46) | 0.10(-0.03,0.23) | 4.86(2.14,7.65) | 0.23(0.06,0.42) |
| Kenya | -0.94(-1.12,-0.76) | -0.62(-0.77,-0.47) | -4.74(-5.64,-3.83) | -1.44(-1.66,-1.21) |
| Kiribati | 0.78(0.73,0.83) | 0.85(0.79,0.92) | 1.99(1.85,2.13) | 1.90(1.75,2.04) |
| Kuwait | -0.17(-0.23,-0.12) | 0.02(-0.05,0.08) | 0.95(-1.20,3.15) | 0.78(-1.22,2.82) |
| Kyrgyzstan | -0.16(-0.24,-0.08) | -0.07(-0.23,0.09) | 21.82(18.31,25.43) | 10.96(9.35,12.58) |
| Lao People's Democratic Republic | -0.72(-0.84,-0.61) | 0.09(0.01,0.18) | 4.01(3.84,4.17) | 1.74(1.60,1.88) |
| Latvia | 0.99(0.84,1.15) | 1.19(1.04,1.34) | 10.48(8.45,12.56) | 9.88(8.21,11.57) |
| Lebanon | -0.40(-0.49,-0.32) | 0.01(-0.05,0.06) | 4.94(4.56,5.32) | 4.33(3.99,4.67) |
| Lesotho | -0.58(-0.68,-0.48) | -0.11(-0.19,-0.04) | -3.35(-3.85,-2.85) | -1.64(-1.89,-1.40) |
| Liberia | -0.26(-0.36,-0.17) | -0.15(-0.24,-0.07) | -0.50(-0.83,-0.16) | -0.19(-0.28,-0.09) |
| Libya | -0.16(-0.24,-0.07) | -0.36(-0.43,-0.30) | 8.95(8.44,9.46) | 1.18(1.04,1.31) |
| Lithuania | 3.91(3.52,4.31) | 3.49(3.24,3.75) | 5.22(3.76,6.69) | 5.15(3.76,6.55) |
| Luxembourg | 0.16(0.06,0.27) | 0.49(0.38,0.61) | 0.94(0.73,1.15) | 0.84(0.65,1.04) |
| Madagascar | -0.40(-0.49, -0.30) | -0.26(-0.34, -0.18) | -7.03(-8.10, -5.95) | -0.87(-1.02, -0.72) |
| Malawi | -0.43(-0.54, -0.33) | -0.23(-0.31, -0.16) | -5.14(-6.11, -4.17) | -0.72(-0.84, -0.60) |
| Malaysia | -0.44(-0.53, -0.35) | 0.10(0.03,0.17) | 0.83(0.57,1.10) | 0.51(0.30,0.72) |
| Maldives | 0.06(-0.02,0.14) | 0.53(0.47,0.59) | 5.10(4.61,5.59) | 4.68(4.23,5.12) |
| Mali | 0.14(0.08,0.20) | 0.41(0.36,0.45) | 1.64(1.44,1.83) | 0.41(0.37,0.46) |
| Malta | 0.56(0.51,0.60) | 0.87(0.82,0.92) | 0.51(0.28,0.75) | 0.42(0.20,0.64) |
| Marshall Islands | -0.76(-0.85, -0.68) | -0.52(-0.59, -0.44) | 0.49(0.15,0.83) | -0.43(-0.61, -0.25) |
| Mauritania | -0.30(-0.41, -0.20) | -0.06(-0.12,0.01) | -0.79(-1.09, -0.49) | -0.11(-0.18, -0.04) |
| Mauritius | 0.01(-0.06,0.09) | 0.39(0.34,0.45) | 8.86(6.07,11.73) | 8.27(5.75,10.84) |
| Mexico | 1.79(1.68,1.90) | 1.59(1.51,1.68) | 2.52(2.29,2.74) | 2.55(2.32,2.78) |
| Micronesia (Federated States of) | -0.51(-0.59, -0.43) | -0.31(-0.37, -0.25) | -0.93(-1.14, -0.73) | -0.84(-0.97, -0.71) |
| Monaco | 0.67(0.61,0.73) | 0.78(0.73,0.84) | 2.49(2.08,2.90) | 2.24(1.87,2.60) |
| Mongolia | -0.14(-0.18, -0.09) | 0.55(0.44,0.65) | 1.02(0.78,1.26) | 0.80(0.58,1.02) |
| Montenegro | -0.40(-0.50, -0.30) | 0.04(-0.07,0.16) | 3.04(2.87,3.21) | 1.70(1.56,1.84) |
| Morocco | -0.51(-0.61, -0.41) | -0.08(-0.14, -0.02) | 8.58(7.98,9.18) | 0.60(0.50,0.70) |
| Mozambique | -0.64(-0.74, -0.54) | -0.01(-0.09,0.06) | -4.48(-5.44, -3.51) | -0.41(-0.51, -0.30) |
| Myanmar | -0.81(-0.93, -0.69) | 0.22(0.12,0.31) | 3.58(3.51,3.65) | 1.90(1.82,1.99) |
| Namibia | -0.46(-0.56, -0.37) | -0.04(-0.11,0.02) | -4.27(-4.81, -3.72) | -2.71(-3.10, -2.32) |
| Nauru | -0.68(-0.77, -0.60) | -0.67(-0.84, -0.50) | -2.41(-3.11, -1.71) | -1.84(-2.29, -1.39) |
| Nepal | -0.29(-0.37, -0.21) | 0.21(0.13,0.28) | 7.83(7.47,8.19) | 3.94(3.60,4.27) |
| Netherlands | 0.95(0.89,1.01) | 1.05(0.98,1.13) | 1.09(0.92,1.26) | 0.98(0.80,1.15) |
| New Zealand | 0.77(0.72,0.81) | 1.06(0.96,1.16) | 0.25(-0.15,0.65) | 0.20(-0.17,0.57) |
| Nicaragua | -0.13(-0.19, -0.07) | 0.12(0.06,0.18) | 3.99(3.58,4.39) | 3.67(3.33,4.02) |
| Niger | -0.10(-0.21,0.00) | -0.06(-0.13,0.02) | -0.42(-0.71, -0.14) | -0.07(-0.14,0.01) |
| Nigeria | -0.64(-0.80, -0.48) | -0.15(-0.26, -0.03) | 1.04(0.69,1.40) | -0.07(-0.19,0.05) |
| Niue | -0.81(-0.90, -0.72) | -0.33(-0.38, -0.27) | -0.83(-0.96, -0.70) | -0.93(-1.03, -0.83) |
| North Macedonia | -0.08(-0.18,0.02) | 0.28(0.18,0.37) | 2.59(2.34,2.85) | 2.44(2.21,2.68) |
| Northern Mariana Islands | -0.17(-0.24, -0.10) | -0.35(-0.45, -0.24) | -0.89(-2.42,0.66) | -0.91(-1.68, -0.13) |
| Norway | 0.77(0.74,0.80) | 1.03(0.94,1.11) | 0.77(0.55,0.99) | 0.70(0.50,0.90) |
| Oman | 0.74(0.64,0.83) | 0.88(0.79,0.97) | 10.66(10.07,11.24) | 7.24(6.73,7.76) |
| Pakistan | -0.04(-0.12,0.04) | 0.36(0.29,0.43) | 5.96(5.57,6.34) | 4.43(4.25,4.61) |
| Palau | -1.02(-1.10, -0.93) | -0.64(-0.72, -0.57) | 2.17(2.02,2.32) | -0.00(-0.04,0.03) |
| Palestine | -0.50(-0.59, -0.40) | -0.13(-0.19, -0.07) | 4.53(4.19,4.88) | 3.85(3.54,4.15) |
| Panama | 0.86(0.74,0.98) | 1.16(1.08,1.24) | 3.78(3.53,4.02) | 3.69(3.46,3.92) |
| Papua New Guinea | -0.47(-0.56, -0.38) | -0.26(-0.34, -0.18) | -1.89(-2.62, -1.16) | -0.57(-0.70, -0.44) |
| Paraguay | -0.38(-0.51, -0.24) | -0.06(-0.16,0.04) | 4.42(4.34,4.51) | 3.41(3.34,3.48) |
| Peru | 0.74(0.68,0.79) | 0.97(0.90,1.04) | 12.29(10.51,14.09) | 10.37(9.08,11.66) |
| Philippines | -0.71(-0.85, -0.56) | -0.25(-0.38, -0.13) | 0.66(0.11,1.22) | 0.47(-0.02,0.96) |
| Poland | 2.81(2.58,3.05) | 2.72(2.55,2.88) | 6.55(5.62,7.49) | 6.33(5.43,7.24) |
| Portugal | 2.32(2.13,2.50) | 2.15(1.96,2.34) | 2.38(2.21,2.55) | 2.42(2.24,2.60) |
| Puerto Rico | 2.35(2.19,2.51) | 2.09(1.94,2.25) | 9.88(6.15,13.73) | 8.76(5.56,12.06) |
| Qatar | -0.31(-0.38, -0.23) | 0.24(0.16,0.31) | 3.03(2.33,3.73) | 0.58(0.48,0.69) |
| Republic of Korea | -0.53(-0.75, -0.30) | 0.35(0.15,0.55) | 0.98(0.30,1.67) | 0.86(0.20,1.53) |
| Republic of Moldova | -0.29(-0.38, -0.20) | 0.02(-0.12,0.16) | 4.28(0.77,7.92) | 3.99(0.90,7.17) |
| Romania | -0.28(-0.36, -0.20) | 0.31(0.19,0.43) | 3.59(3.36,3.81) | 3.42(3.20,3.64) |
| Russian Federation | 2.75(2.46,3.04) | 2.28(1.98,2.58) | 9.60(8.41,10.79) | 9.21(8.07,10.36) |
| Rwanda | -0.50(-0.60, -0.39) | -0.05(-0.14,0.04) | -6.37(-7.55,-5.17) | -0.78(-0.98,-0.59) |
| Saint Kitts and Nevis | 2.17(2.06,2.28) | 1.94(1.84,2.04) | 12.62(9.02,16.34) | 11.48(8.48,14.56) |
| Saint Lucia | 2.37(2.24,2.51) | 2.03(1.93,2.13) | 10.73(7.27,14.30) | 9.83(6.80,12.93) |
| Saint Vincent and the Grenadines | 1.50(1.39,1.60) | 1.37(1.30,1.45) | 8.39(6.13,10.70) | 7.80(5.75,9.90) |
| Samoa | -0.80(-0.89, -0.72) | -0.40(-0.46, -0.34) | -1.01(-1.28,-0.74) | -0.88(-1.02,-0.74) |
| San Marino | -1.14(-1.34, -0.94) | -0.61(-0.78, -0.44) | 0.21(-0.18,0.61) | 0.14(-0.20,0.48) |
| Sao Tome and Principe | 1.50(1.40,1.60) | 1.48(1.37,1.58) | 3.82(3.61,4.02) | 1.58(1.47,1.69) |
| Saudi Arabia | -0.44(-0.53, -0.35) | -0.05(-0.09, -0.01) | 5.52(4.83,6.22) | 4.38(3.89,4.87) |
| Senegal | -0.29(-0.39, -0.20) | -0.11(-0.18, -0.03) | 0.39(0.09,0.69) | -0.10(-0.18, -0.03) |
| Serbia | -0.81(-0.96, -0.67) | -0.26(-0.40, -0.12) | 2.67(2.52,2.83) | 2.14(2.00,2.29) |
| Seychelles | 0.53(0.46,0.60) | 0.67(0.61,0.73) | 3.24(2.66,3.82) | 1.88(1.62,2.15) |
| Sierra Leone | -0.20(-0.30, -0.09) | -0.17(-0.24, -0.09) | 0.01(-0.35,0.37) | -0.17(-0.25, -0.09) |
| Singapore | 0.22(0.18,0.27) | 0.88(0.83,0.94) | 0.12(-0.16,0.40) | 0.01(-0.26,0.27) |
| Slovakia | -0.23(-0.32, -0.13) | 0.26(0.18,0.35) | 2.82(2.54,3.11) | 2.58(2.34,2.83) |
| Slovenia | -1.44(-1.70, -1.18) | -0.91(-1.11, -0.70) | 2.20(1.84,2.56) | 1.92(1.56,2.28) |
| Solomon Islands | -0.45(-0.54, -0.35) | -0.31(-0.39, -0.24) | -0.99(-1.68, -0.29) | -0.52(-0.66, -0.38) |
| Somalia | -0.24(-0.34, -0.15) | -0.33(-0.41, -0.25) | -7.70(-8.66, -6.73) | -0.60(-0.70, -0.50) |
| South Africa | -0.52(-0.68, -0.36) | -0.24(-0.36, -0.11) | -4.24(-4.64, -3.84) | -2.57(-2.88, -2.26) |
| South Sudan | -0.18(-0.29, -0.07) | -0.35(-0.43, -0.28) | -6.64(-7.62, -5.65) | -0.89(-0.97, -0.81) |
| Spain | 1.52(1.35,1.69) | 1.42(1.26,1.58) | 1.91(1.60,2.21) | 1.71(1.43,2.00) |
| Sri Lanka | -0.28(-0.33, -0.22) | 0.35(0.30,0.40) | 8.60(7.59,9.61) | 7.43(6.56,8.31) |
| Sudan | -0.50(-0.60, -0.41) | 0.02(-0.04,0.08) | 9.47(8.75,10.20) | 0.51(0.42,0.60) |
| Suriname | 1.53(1.44,1.61) | 1.49(1.41,1.57) | 10.94(8.15,13.80) | 9.73(7.36,12.15) |
| Sweden | 0.99(0.92,1.05) | 0.85(0.60,1.10) | 1.10(0.83,1.36) | 0.97(0.72,1.22) |
| Switzerland | 0.01(-0.02,0.04) | 0.28(0.25,0.31) | 0.42(0.21,0.63) | 0.31(0.09,0.52) |
| Syrian Arab Republic | -0.47(-0.57,-0.37) | -0.15(-0.21,-0.08) | 6.37(5.91,6.84) | 0.52(0.44,0.59) |
| Taiwan (Province of China) | -0.54(-1.29,0.22) | -0.04(-0.73,0.66) | 1.40(0.93,1.87) | 1.31(0.87,1.76) |
| Tajikistan | -0.39(-0.47,-0.30) | -0.23(-0.41,-0.04) | 6.10(5.43,6.78) | 0.97(0.71,1.23) |
| Thailand | -0.52(-0.63,-0.42) | 0.02(-0.05,0.09) | 3.77(3.45,4.10) | 2.47(2.18,2.75) |
| Timor-Leste | -0.67(-0.79,-0.54) | 0.01(-0.03,0.06) | 2.94(2.77,3.10) | 1.11(1.05,1.17) |
| Togo | -0.29(-0.39,-0.20) | -0.18(-0.26,-0.10) | -0.69(-0.95,-0.43) | -0.22(-0.30,-0.14) |
| Tokelau | -0.71(-0.79,-0.63) | -0.26(-0.32,-0.19) | -0.55(-0.79,-0.30) | -0.77(-0.91,-0.63) |
| Tonga | -0.59(-0.68,-0.50) | -0.29(-0.37,-0.21) | -0.63(-0.98,-0.28) | -0.67(-0.84,-0.51) |
| Trinidad and Tobago | 1.18(1.03,1.33) | 1.39(1.28,1.51) | 10.93(7.27,14.71) | 9.83(6.66,13.11) |
| Tunisia | -0.51(-0.60,-0.41) | -0.03(-0.09,0.04) | 6.66(6.28,7.05) | 0.72(0.65,0.79) |
| Turkey | 1.47(1.38,1.55) | 1.46(1.39,1.53) | 4.17(3.89,4.46) | 4.01(3.73,4.29) |
| Turkmenistan | -0.05(-0.09,-0.00) | 0.56(0.39,0.73) | 1.05(-0.79,2.91) | 0.54(0.35,0.74) |
| Tuvalu | -0.86(-0.96,-0.77) | -0.48(-0.56,-0.40) | 0.36(0.15,0.56) | -0.51(-0.64,-0.37) |
| Uganda | -0.60(-0.70,-0.50) | -0.10(-0.16,-0.03) | -3.32(-4.11,-2.52) | -0.52(-0.63,-0.40) |
| Ukraine | -0.40(-0.52, -0.28) | -0.20(-0.37, -0.03) | 4.47(3.64,5.30) | 3.94(3.23,4.66) |
| United Arab Emirates | 0.19(0.15,0.24) | -0.01(-0.07,0.05) | 6.15(5.25,7.06) | 5.58(4.73,6.42) |
| United Kingdom | 1.75(1.66,1.84) | 1.69(1.57,1.81) | 1.65(1.26,2.03) | 1.42(1.06,1.79) |
| United Republic of Tanzania | -0.50(-0.61, -0.40) | -0.08(-0.16,0.01) | -5.61(-6.49,-4.71) | -0.79(-0.96,-0.63) |
| United States of America | 1.18(1.06,1.30) | 1.54(1.27,1.80) | 0.94(0.49,1.40) | 0.85(0.43,1.26) |
| United States Virgin Islands | 2.58(2.35,2.82) | 2.20(1.94,2.45) | 3.24(2.14,4.34) | 3.04(2.02,4.08) |
| Uruguay | 2.16(1.89,2.43) | 2.27(2.04,2.50) | 7.68(5.70,9.70) | 7.33(5.48,9.22) |
| Uzbekistan | -0.55(-0.63, -0.47) | 0.02(-0.12,0.16) | 10.34(9.67,11.02) | 0.15(-0.01,0.30) |
| Vanuatu | -0.50(-0.59, -0.41) | -0.32(-0.39, -0.24) | -0.79(-1.37,-0.20) | -0.52(-0.64,-0.39) |
| Venezuela (Bolivarian Republic of) | 1.57(1.48,1.66) | 1.34(1.24,1.45) | 3.74(3.14,4.35) | 3.60(3.00,4.20) |
| Viet Nam | -0.54(-0.63, -0.45) | 0.23(0.16,0.30) | 3.14(2.97,3.32) | 2.11(2.04,2.19) |
| Yemen | -0.36(-0.46, -0.26) | -0.13(-0.20, -0.06) | 10.72(9.97,11.48) | 0.35(0.29,0.41) |
| Zambia | -0.66(-0.78, -0.55) | -0.14(-0.22, -0.06) | -5.52(-6.55,-4.47) | -0.97(-1.18,-0.76) |
| Zimbabwe | -0.07(-0.13, -0.01) | -0.26(-0.33, -0.18) | -0.30(-0.81,0.23) | -0.33(-0.82,0.16) |

MND, motor neuron disease; DALYs, disability-adjusted life years; EAPCs, estimated annual percentage changes.

Table S7 Forecasted global cases and age-standardized rates of elderly motor neuron disease from 2021 to 2040

| **Location** | **Cases** | | | **ASR** | | |
| --- | --- | --- | --- | --- | --- | --- |
|  | **2021** | **2030** | **2040** | **2021** | **2030** | **2040** |
| **Incident** |  |  |  |  |  |  |
| Global | 28048.54 | 35480.20 | 42025.58 | 3.64 | 3.48 | 3.15 |
| High SDI | 18860.25 | 9393.24 | 4109.05 | 3.08 | 1.32 | 0.51 |
| High-middle SDI | 4773.26 | 2769.06 | 2354.43 | 0.77 | 0.6 | 0.41 |
| Middle SDI | 2836.59 | 2424.07 | 2139.15 | 0.21 | 0.14 | 0.09 |
| Low-middle SDI | 1093.97 | 3891.99 | 7607.26 | 0.28 | 0.38 | 0.54 |
| Low SDI | 374.32 | 1420.53 | 4178.75 | 0.46 | 0.82 | 1.58 |
| **Prevalent** |  |  |  |  |  |  |
| Global | 88264.99 | 108606.42 | 124014.6 | 11.46 | 10.66 | 9.29 |
| High SDI | 62760.29 | 30349.32 | 12833.09 | 10.23 | 4.26 | 1.6 |
| High-middle SDI | 16218.74 | 9370.35 | 7439.28 | 2.62 | 2.04 | 1.29 |
| Middle SDI | 6299.71 | 5635.33 | 5272.42 | 0.46 | 0.33 | 0.23 |
| Low-middle SDI | 2133.68 | 7748.07 | 15462.52 | 0.54 | 0.76 | 1.09 |
| Low SDI | 572.91 | 2200.02 | 6504.27 | 0.7 | 1.28 | 2.45 |
| **Death** |  |  |  |  |  |  |
| Global | 25348.69 | 34392.54 | 44432.26 | 3.29 | 3.37 | 3.32 |
| High SDI | 17479.64 | 8589.17 | 3705.32 | 2.85 | 1.21 | 0.46 |
| High-middle SDI | 5119.27 | 3817.56 | 4362.6 | 0.83 | 0.83 | 0.75 |
| Middle SDI | 1833.17 | 1905.69 | 2086.6 | 0.13 | 0.11 | 0.09 |
| Low-middle SDI | 343.91 | 1876.33 | 5887.35 | 0.09 | 0.18 | 0.41 |
| Low SDI | 11.33 | 100.12 | 744.22 | 0.01 | 0.06 | 0.28 |
| **DALYs** |  |  |  |  |  |  |
| Global | 461092.47 | 610710.78 | 778474.95 | 59.87 | 59.94 | 58.32 |
| High SDI | 327916.3 | 161785.78 | 70644.92 | 53.47 | 22.71 | 8.8 |
| High-middle SDI | 95841.31 | 71924.37 | 83861.28 | 15.47 | 15.64 | 14.5 |
| Middle SDI | 36056.71 | 37828.25 | 44417.02 | 2.63 | 2.22 | 1.96 |
| Low-middle SDI | 6679.59 | 34066.36 | 101871.41 | 1.69 | 3.34 | 7.17 |
| Low SDI | 352.81 | 1819.05 | 7498.31 | 0.43 | 1.06 | 2.83 |

SDI, socio-demographic index; ASR, age-standardized rates; DALYs, disability-adjusted life years.
